# Switchgrass (*Panicum virgatum* L.) promoters for green tissue-specific expression of the *MYB4* transcription factor for reduced-recalcitrance transgenic switchgrass

**DOI:** 10.1186/s13068-018-1119-7

**Published:** 2018-04-24

**Authors:** Wusheng Liu, Mitra Mazarei, Rongjian Ye, Yanhui Peng, Yuanhua Shao, Holly L. Baxter, Robert W. Sykes, Geoffrey B. Turner, Mark F. Davis, Zeng-Yu Wang, Richard A. Dixon, C. Neal Stewart

**Affiliations:** 1 Department of Plant Sciences, University of Tennessee, Knoxville, TN USA; 2 Department of Horticultural Science, North Carolina State University, Raleigh, NC USA; 3 National Renewable Energy Laboratory, Golden, CO USA; 4 Noble Research Institute, Ardmore, OK USA; 5 BioDiscovery Institute and Department of Biological Sciences, University of North Texas, Denton, TX USA; 6 BioEnergy Science Center (BESC), Oak Ridge National Laboratory, Oak Ridge, TN USA

**Keywords:** Green tissue-specific promoter, *PvLhcb*, *PvPEPC*, *PvPsbR*, *PvMYB4*, Transgenic rice, Transgenic switchgrass

## Abstract

**Background:**

Genetic engineering of switchgrass (*Panicum virgatum* L.) for reduced cell wall recalcitrance and improved biofuel production has been a long pursued goal. Up to now, constitutive promoters have been used to direct the expression of cell wall biosynthesis genes toward attaining that goal. While generally sufficient to gauge a transgene’s effects in the heterologous host, constitutive overexpression often leads to undesirable plant phenotypic effects. Green tissue-specific promoters from switchgrass are potentially valuable to directly alter cell wall traits exclusively in harvestable aboveground biomass while not changing root phenotypes.

**Results:**

We identified and functionally characterized three switchgrass green tissue-specific promoters and assessed marker gene expression patterns and intensity in stably transformed rice (*Oryza sativa* L.), and then used them to direct the expression of the switchgrass *MYB4* (*PvMYB4*) transcription factor gene in transgenic switchgrass to endow reduced recalcitrance in aboveground biomass. These promoters correspond to photosynthesis-related light-harvesting complex II chlorophyll-a/b binding gene (*PvLhcb*), phosphoenolpyruvate carboxylase (*PvPEPC*), and the photosystem II 10 kDa R subunit (*PvPsbR*). Real-time RT-PCR analysis detected their strong expression in the aboveground tissues including leaf blades, leaf sheaths, internodes, inflorescences, and nodes of switchgrass, which was tightly up-regulated by light. Stable transgenic rice expressing the *GUS* reporter under the control of each promoter (756–2005 bp in length) further confirmed their strong expression patterns in leaves and stems. With the exception of the serial promoter deletions of *PvLhcb*, all *GUS* marker patterns under the control of each 5′-end serial promoter deletion were not different from that conveyed by their respective promoters. All of the shortest promoter fragments (199–275 bp in length) conveyed strong green tissue-specific *GUS* expression in transgenic rice. *PvMYB4* is a master repressor of lignin biosynthesis. The green tissue-specific expression of *PvMYB4* via each promoter in transgenic switchgrass led to significant gains in saccharification efficiency, decreased lignin, and decreased S/G lignin ratios. In contrast to constitutive overexpression of *PvMYB4*, which negatively impacts switchgrass root growth, plant growth was not compromised in green tissue-expressed *PvMYB4* switchgrass plants in the current study.

**Conclusions:**

Each of the newly described green tissue-specific promoters from switchgrass has utility to change cell wall biosynthesis exclusively in aboveground harvestable biomass without altering root systems. The truncated green tissue promoters are very short and should be useful for targeted expression in a number of monocots to improve shoot traits while restricting gene expression from roots. Green tissue-specific expression of *PvMYB4* is an effective strategy for improvement of transgenic feedstocks.

**Electronic supplementary material:**

The online version of this article (10.1186/s13068-018-1119-7) contains supplementary material, which is available to authorized users.

## Background

Switchgrass (*Panicum virgatum* L.) is a C_4_ warm season perennial forage grass and a leading lignocellulosic feedstock for renewable bioenergy production in the United States [1]. It is well adapted to eastern North America with a wide range of genomic variation, high biomass yield, efficient use of water and nutrients, and resilience to biotic and abiotic stresses [2]. Genetic engineering of switchgrass has been performed to increase biomass [3–8], modify flowering [9, 10], improve plant architecture [11], reduce cell wall recalcitrance (i.e., resistance of cell wall to deconstruction and conversion into biofuels) [11–19], and improve water and nutrition use efficiency [20].

So far, the number of promoters used for switchgrass transformation and genetic engineering has been very limited. The list consists of constitutive promoters such as the cauliflower mosaic virus (CaMV) 35S promoter [3, 20–22], the rice actin 1 (*OsAct1*) promoter [23], and the ubiquitin promoters from maize (*Ubi*-*1*) [5–8, 10, 11, 14, 16–19, 24–26], rice (*rubi2* and *rubi3*) [27, 28], and switchgrass (*PvUbi1* and *PvUbi2*) [29]. Constitutive promoters allow high levels of constant gene expression in all tissues at all developmental stages, and permit first-order analysis of phenotypes conferred by a transgene. Such continuous high level of expression of some transgenes may cause side effects to the host plants, such as homology-dependent gene silencing [30, 31], unintended impacts on growth and development [32–34], and abnormal morphology [32, 35–40]. A good example is when switchgrass *MYB4* (*PvMYB4*) gene was overexpressed in transgenic switchgrass under the control of *ZmUbi1* promoter [12, 17]. The best *PvMYB4* overexpression line during a two-year field experiment produced 32% more biofuel and 63% more biomass than the non-transgenic switchgrass, which represents a doubling of biofuel production per hectare and the highest gain among all of the reported field-grown genetically modified feedstocks [12]. However, since *PvMYB4* encodes an R2–R3-type transcription factor that acts as a negative regulator of many lignin biosynthetic genes, it was observed that negative growth effects and yield penalties were associated with the high expression levels of the transgenic *PvMYB4* in several of the field-grown switchgrass overexpression lines [12]. The best *PvMYB4* overexpression lines in terms of low lignin content and high biofuel and biomass production had low-to-moderate ectopic expression levels. The high-level ectopic expression lines exhibited reduced tiller height, plant width (i.e., the diameter at the mid-section of each whole plant; spread of tillers), and tiller numbers [12, 17], and did not survive the first winter in the field [12]. Moreover, the high-level ectopic expression lines also suffered from weak, undeveloped, or diminished (so-called mushy) root systems, indicating a disruptive effect of *PvMYB4* overexpression on the root system [12]. Thus, there is an urgent need for green tissue-specific promoters to limit *PvMYB4* overexpression to the aboveground tissues, where the harvestable biomass is produced each growing season.

Multiple green tissue-specific promoters have been well characterized in some monocot species such as maize and rice. These include the promoters of maize phosphoenolpyruvate carboxylase (*PEPC*) [41–43], pyruvate orthophosphate dikinase (*PPDK*) [44] and the small subunit of ribulose-1, 5-bisphosphate carboxylase/oxygenase (*rbcS*) [43], rice light-harvesting complex II chlorophyll-a/b binding gene (*Lhcb*; also known as *Cab*) [45–47], *rbcS* [48], *Leaf Panicle 2* (*LP2*) [49], *D54O* [50], and *DX1* [51]. These genes are tightly light-inducible in green tissues, and their proteins are involved in photosynthesis that converts light energy into sugar. For example, *Lhcb* encodes a protein that functions in photosystems I and II by binding to chlorophyll. *PEPC* in C_4_ plants encodes a cytosolic enzyme that catalyzes the conversion of phosphoenolpyruvate and bicarbonate to four carbon acid oxaloacetate and inorganic phosphate, even though its primary function in C_3_ plants is anaplerotic by replenishing the tricarboxylic acid cycle with intermediates [52]. *PsbR* encodes the subunit R of the photosystem II 10 kDa polypeptide.

In the present study, we identified and functionally characterized three switchgrass green tissue-specific promoters (i.e., *PvLhcbp*, *PvPEPCp,* and *PvPsbRp*; *p* stands for promoter) in endogenous plant tissues as well as in transgenic rice. We characterized the functions of truncations of each promoter. We also fused the green tissue promoters with *PvMYB4* to study the effects of targeted green tissue expression of this transcriptional repressor in transgenic switchgrass on plant growth and sugar release.

## Results

### Sequence analysis and expression patterns of three green tissue-specific genes in switchgrass

Using the rice green tissue-specific genes *OsLhcb*, *OsPEPC,* and *OsPsbR* as the query sequences, the BlastP search for their homologous sequences in the switchgrass genome returned 11, 8, and 4 candidate sequences, respectively, with high amino acid sequence similarities (Additional file 1: Figs. S1–S3). The cDNA sequence analysis indicated that all of these candidate sequences had the same exon/intron structures as their rice homologs (Additional file 1: Figs. S4–S6) except 6 out of the 11 switchgrass *Lhcb* (*PvLhcb*) sequences, which contained a single intron at variable positions while their rice homologs were intronless (Additional file 1: Fig. S4).

**Fig. 1:**
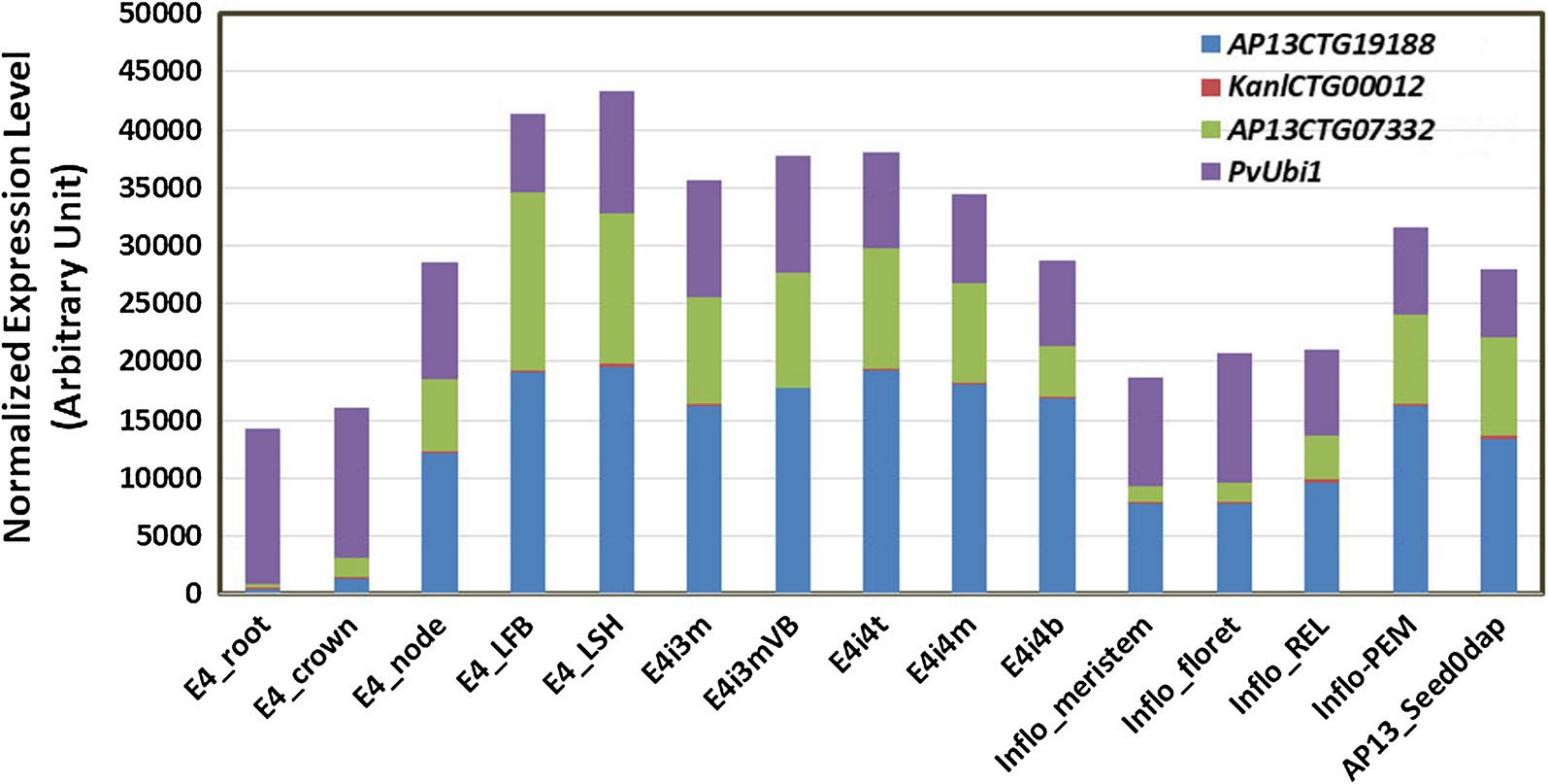
In silico expression profiles (fold expression) of the unitranscripts of the three switchgrass green tissue-specific genes in different tissues. The cDNA sequences of the three switchgrass green tissue-specific genes as well as the *PvUbi1* gene, i.e., *Pavirv00047797m, Pavirv00033161m, Pavirv00009702m*, and *Pavirv00038913m*, were used to blast the Noble Foundation switchgrass gene expression atlas PviUT V1.2 (http://switchgrassgenomics.noble.org/). *AP13CTG19188*, *KanlCTG00012*, *AP13CTG07332*, and *KanlCTG00705* were the unitranscripts of *PvLhcb, PvPEPC*, *PvPsbR*, and *PvUbi1,* respectively. E4-root, whole root system of E4 stage plant; E4-crown, whole crown of E4 stage plant; E4-node, pooled nodes of E4 the tiller; E4-LFB, pooled leaf blade from E4 tiller; E4-LSH, pooled leaf sheath of the E4 tiller; E4i3m, middle 1/5 fragment of internode 3; E4i3mVB, vascular bundle isolated from 1/5 fragment of internode 3; E4i4t, top 1/5 fragment of internode 4; E4i4m, middle 1/5 fragment of internode 4; E4i4b, bottom 1/5 fragment of internode 4; inflo-meristem, inflorescence of rachis, primary and secondary branch meristem initiation stages 0.5–3.0 mm; inflo-floret, inflorescence of glume and floret development stages 10–20 mm; Inflo-REL, inflorescence 50–150 mm; Inflo-PEM, inflorescence > 200 mm

The 11, 8, and 4 switchgrass candidate sequences, which, respectively, corresponded to *OsLhcb*, *OsPEPC*, and *OsPsbR*, had the highest sequence identity to 8, 6, and 3 Noble Foundation Gene Atlas unitranscript entries, respectively (Additional file 1: Figs. S7–S9). In silico expression analysis revealed that these unitranscript entries showed highly variable expression levels in different switchgrass tissues (Additional file 1: Figs. S7–S9). The entries of *PvLhcb* and the switchgrass *PsbR* (*PvPsbR*) were mainly expressed in the aboveground tissues with *AP13CTG19188* (i.e., the entry of *Pavirv00047797m* and *Pavirv00024895m*) and *AP13CTG07332* (i.e., the entry of *Pavirv00009702m*) being the strongest expressed entries of both genes, respectively (Additional file 1: Figs. S7, S9). Both entries were highly expressed in leaves, internodes, nodes, inflorescences that were taller than 200 mm in length, and whole flowers. They were moderately expressed in inflorescences of rachis, primary and secondary branch meristem of 0.5–3.0 mm in length at the initiation stage, inflorescences of the glume and floret developmental stages of 10–20 mm in length, and inflorescences of 50–150 mm in length. They were weakly expressed in the whole crown of E4 stage plant, and minimally expressed in roots (Fig. 1; Additional file 1: Figs. S7, S9). Unexpectedly, all of the entries, including *KanlCTG00012*, which is the entry of *Pavirv00033161m*, of the switchgrass *PEPC* (*PvPEPC*) had extremely low expression levels in different tissues (Additional file 1: Fig. S8). Based on the sequence similarities, gene structure, and in silico expression patterns, *Pavirv00047797m*, *Pavirv00033161m,* and *Pavirv00009702m* were selected as the potential switchgrass homologs of the rice green tissue-specific genes *OsLhcb*, *OsPEPC*, and *OsPsbR* for further analysis and named *PvLhcb*, *PvPEPC,* and *PvPsbR* hereafter, respectively (Fig. 1). Those genes were the targets for promoter characterization and manipulation.

**Fig. 2:**
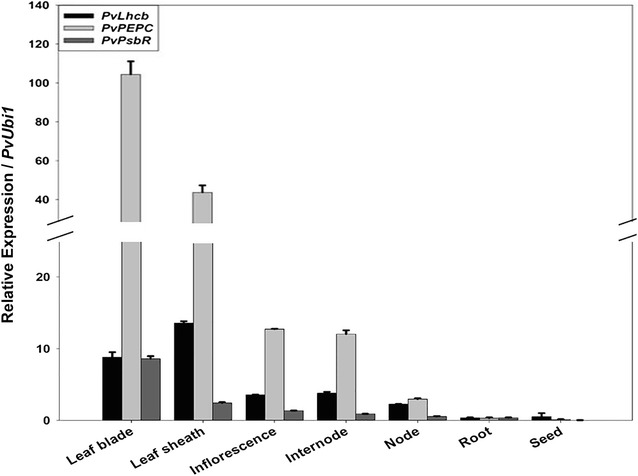
Endogenous relative expression levels of the three switchgrass green tissue-specific genes in different tissues measured by real-time RT-PCR. RNA was extracted from switchgrass cv. ‘Alamo’ grown in the greenhouse at 28 °C under 16-h day/8-h night photoperiods (390 μE/m^2^/s) at the R1 growth stage. Relative quantification was performed using the standard curve method with *PvUbi1* as the internal control gene. Bars represent the mean values of three independent replicates ± standard errors (vertical bars)

**Fig. 3:**
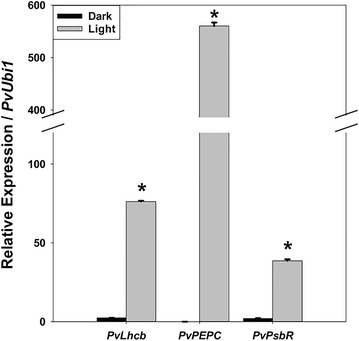
Light induction of the relative expression of the three switchgrass green tissue-specific genes in the shoots of 3-week-old switchgrass cv. ‘Alamo’ seedlings measured by real-time RT-PCR. RNA was extracted from the shoots of switchgrass seedlings grown in the greenhouse at 28 °C under 16-h day/8-h night photoperiods (390 μE/m^2^/s). Relative quantification was performed using the standard curve method with *PvUbi1* as the internal control gene. Bars represent the mean values of three independent replicates ± standard errors (vertical bars). Bars with asterisk are significantly different from the dark treatments at *p *≤ 0.05 as calculated by *t* test. Dark, 3-week-old seedlings growing under 24-h dark conditions at 28 °C; light, 3-week-old seedlings growing under 16/8-h light/dark cycles at 28 °C

Real-time RT-PCR analysis demonstrated that the expression of *PvLhcb*, *PvPEPC,* and *PvPsbR* were highest in leaf blade and leaf sheath, followed by inflorescence, internode, and node, but marginally expressed in root and seed of switchgrass plants grown in the greenhouse at 28 °C under 16-h day/8-h night photoperiods (390 μE/m^2^/s) at the R1 growth stage (Fig. 2). The relative expression levels of each gene in root and seed were comparable to each other. However, the relative expression levels of *PvPEPC* in most of the aboveground tissues were much higher than that of the *PvLhcb* and *PvPsbR* genes. When compared to the *PvUbi1* expression levels, *PvPEPC* had 104.4-, 43.7-, 12.7-, and 12.0-fold higher expression in the leaf blade, leaf sheath, inflorescence, and internode, respectively (Fig. 2). *PvLhcb* and *PvPsbR* in the four aboveground tissues had 3.8–8.8 and 0.9–8.6 times higher expression than that of *PvUbi1, respectively* (Fig. 2). Moreover, exposure to light with an intensity of 390 μE/m^2^/s enhanced the expression of *PvLhcb*, *PvPEPC*, and *PvPsbR* in the switchgrass shoot to 38.6, 560.2, and 76.2 times higher than *PvUbi1*, respectively (Fig. 3). In contrast, the three genes had minimal expression levels when grown in the dark in comparison to the *PvUbi1* (Fig. 3). As a result, we concluded these three switchgrass genes are green tissue-specific and highly light-inducible, and their promoters were used for further functional analysis.

### GUS expression driven by each green tissue-specific promoter in stable transgenic rice

The promoter sequences of the *PvLhcb*, *PvPEPC*, and *PvPsbR* genes (i.e., *PvLhcbp*, *PvPEPCp*, and *PvPsbRp*) obtained from the switchgrass genome were 764, 1878, and 2009 bp in length, respectively (Additional file 1: Figs. S10–S12). The start codon and the translation termination codon of *PvLhcb* were located 764 and 1153 bp, respectively, downstream from the beginning of the 5′-end of the contig165429 (Additional file 1: Fig. S10). The start codon and the translation termination codon of *PvPEPC* were located 1977 and 4863 bp, respectively, downstream of the start codon of its nearest upstream gene (*Pavir.J09521.1*; unknown function) that was reversely orientated on the contig12474 (Additional file 1: Fig. S11). The start codon and the translation termination codon of *PvPsbR* were located 4350 and 4746 bp, respectively, downstream of the start codon of its nearest upstream gene (*Pavir.Fa02068.1*; a pentatricopeptide repeat-containing protein) that was reversely orientated on Chr06a (Additional file 1: Fig. S12).

**Fig. 4:**
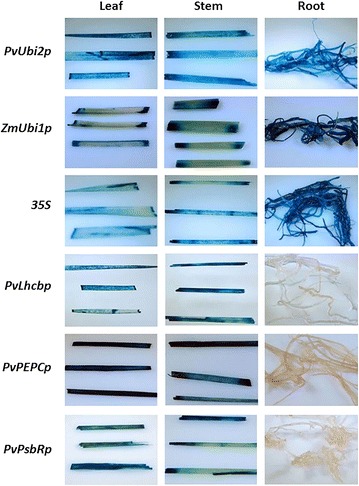
Histochemical GUS analysis of leaf, stem, and root of T0 stable transgenic rice containing each of the three green tissue-specific promoters at the seedling stage. Stable transgenic rice containing each of the promoters of *PvUbi2*, *ZmUbi1,* and *CaMV 35S* were used as the positive controls. The histochemical GUS assay was conducted on at least 10 transgenic lines with the representatives being shown

The GUS activities under the control of each of the three green tissue-specific promoters were examined in T0 transgenic rice at two different developmental stages, i.e., the seedling stage and the heading stage. Histochemical GUS analysis of transgenic rice plants at the seedling stage revealed ectopic constitutive GUS expression in leaves, stems, and roots of the three positive controls (Fig. 4). The transgenic rice expressing GUS under the control of *PvLhcbp*, *PvPEPCp*, or *PvPsbRp* had strong GUS expression in leaves and stems, which was comparable to that in the three positive controls (Fig. 4). However, GUS expression driven by each green tissue-specific promoter was only marginally detectable in roots (Fig. 4). Thus, we concluded that *PvLhcbp*, *PvPEPCp*, or *PvPsbRp* have green tissue-specific activity in transgenic rice.

**Fig. 5:**
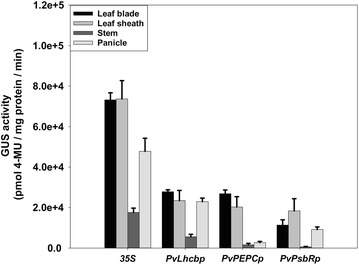
Quantitative fluorometric GUS analysis of leaf blade, leaf sheath, stem, and panicles of T0 stable transgenic rice containing each of the three green tissue-specific promoters at the heading stage. The rice plants were grown in the greenhouse at 25–29 °C under 12-h photoperiods (390 μE/m^2^/s). Stable transgenic rice containing the promoter of *CaMV 35S* was used as positive control. The fluorometric GUS assay was conducted on at least 10 transgenic lines at the heading stage with the representatives being shown

A more sensitive fluorometric GUS assay was then used to measure GUS activities in leaf blade, leaf sheath, stems, and panicles of T0 transgenic rice plants at the heading stage. The root was not included here in the fluorometric GUS analysis due to a slight expression level of each of the three green tissue-specific genes in the roots of non-transgenic switchgrass (Fig. 2) and GUS-reporter transgenic rice (Fig. 4). Transgenic rice containing the *35S::GUS*-positive control showed strong *GUS* expression in leaf blade, leaf sheath, and panicles with a relatively moderate *GUS* expression in stems (Fig. 5). Transgenic rice containing *GUS* driven by each of the three promoters showed moderate *GUS* expression in leaf blades and leaf sheath of about 15.5–38.8% of the *35S* activities in both tissues (Fig. 5). All of the three promoters exhibited relatively low GUS activities in stems. In panicles, the GUS activities driven by *PvLhcbp* and *PvPsbRp* were relatively moderate, whereas that driven by *PvPEPCp* was low in panicles (Fig. 5).

### GUS expression driven by 5′-end serial deletions of each green tissue-specific promoter in stable transgenic rice

**Fig. 6:**
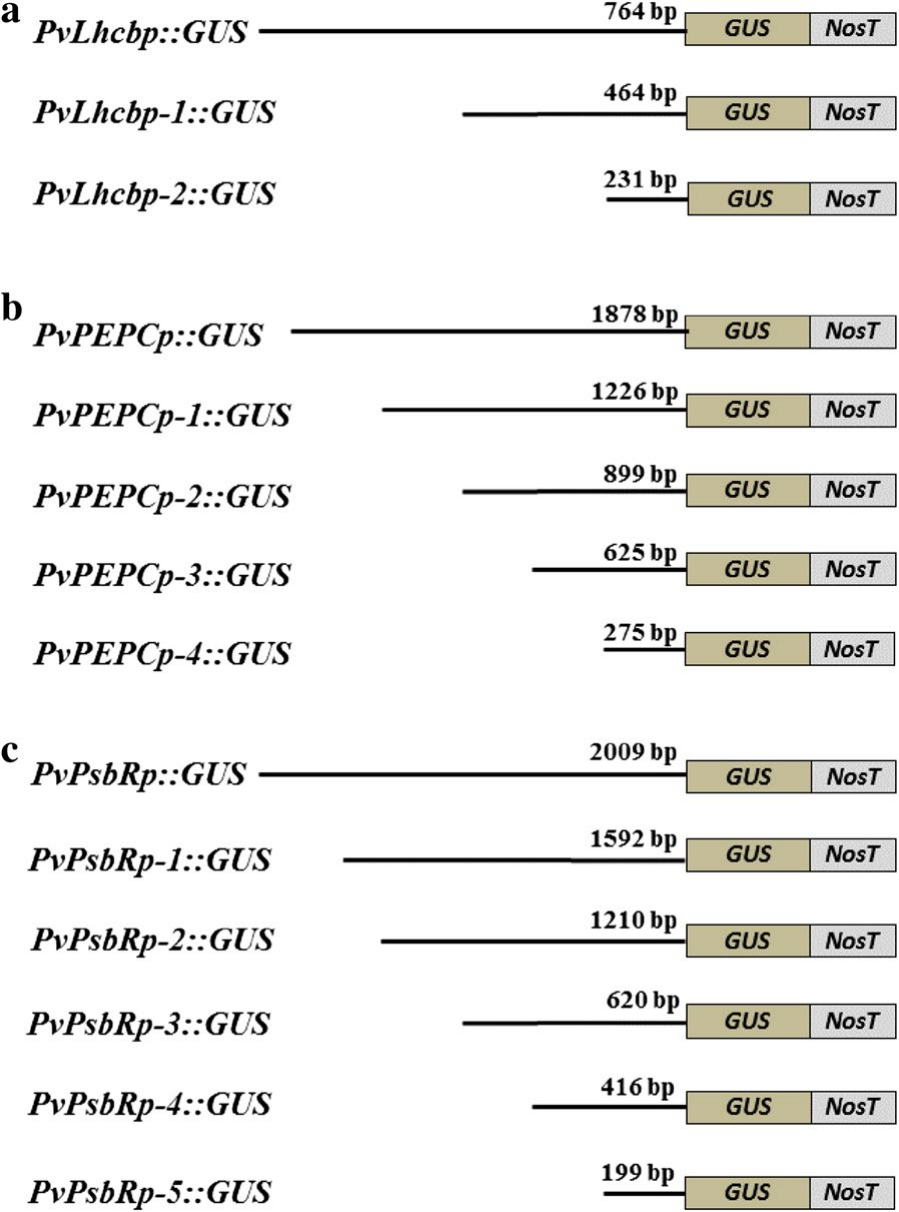
Scheme of the *promoter::GUS* fusion constructs used in stable rice transformation experiments. **a** The *PvLhcb* promoter and its serial deletions driving *GUS* expression. **b** The *PvPEPC* promoter and its serial deletions driving *GUS* expression. **c** The *PvPsbR* promoter and its serial deletions driving *GUS* expression. Lines represent promoters with promoter length (base pairs; bp) being indicated above each line. NosT, Nos terminator

**Fig. 7:**
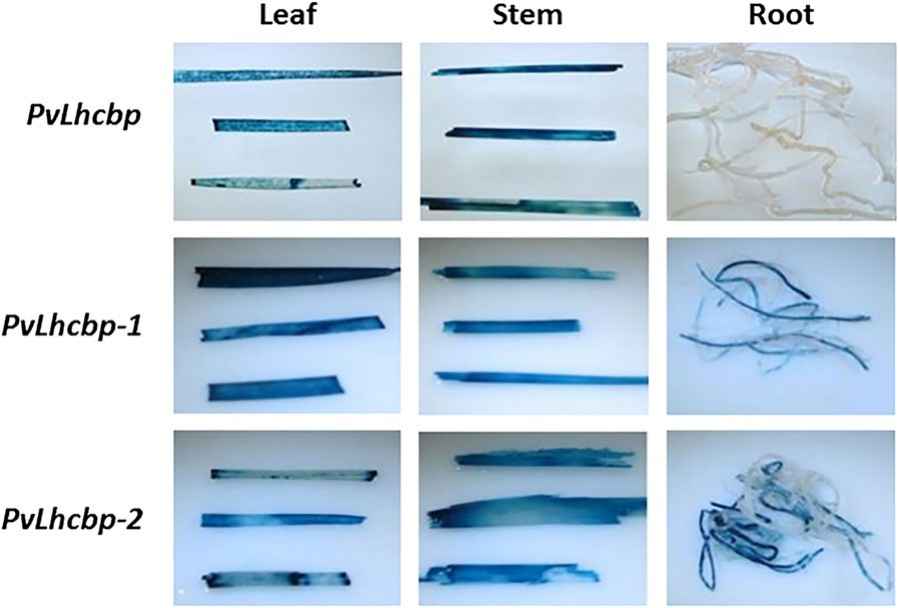
Histochemical GUS analysis of leaf, stem, and root of T0 stable transgenic rice containing each of the serial promoter deletions of *PvLhcbp*. The histochemical GUS assay was conducted on at least 10 transgenic lines of each construct at the seedling stage with one representative line of each construct being shown

**Fig. 8:**
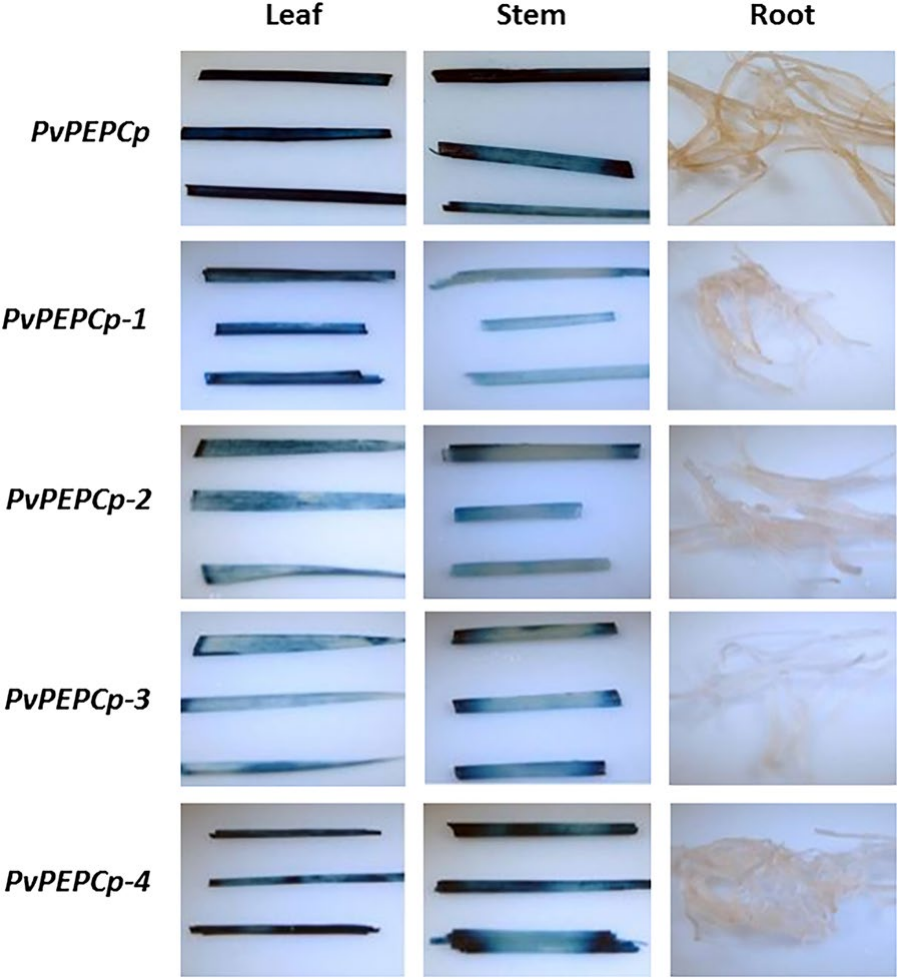
Histochemical GUS analysis of leaf, stem, and root of T0 stable transgenic rice containing each of the serial promoter deletions of *PvPEPCp*. The histochemical GUS assay was conducted on at least 10 transgenic lines of each construct at the seedling stage with one representative line of each construct being shown

**Fig. 9:**
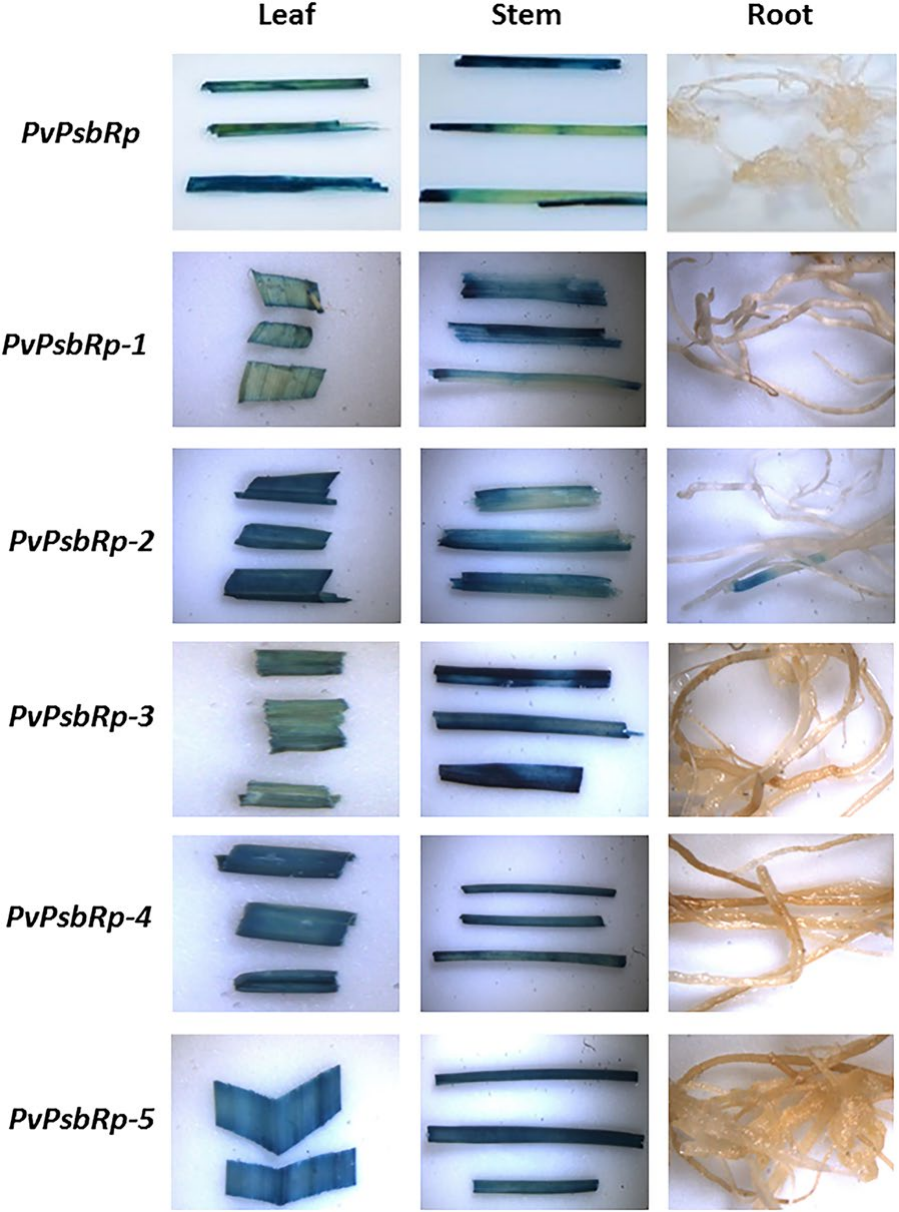
Histochemical GUS analysis of leaf, stem, and root of T0 stable transgenic rice containing each of the serial promoter deletions of *PvPsbRp*. The histochemical GUS assay was conducted on at least 10 transgenic lines of each construct at the seedling stage with one representative line of each construct being shown

The 5′-end serial promoter deletions (Fig. 6) were generated to assess the functionality of various portions, including the core promoter regions, of *PvLhcbp*, *PvPEPCp*, and *PvPsbRp*. Histochemical and fluorometric GUS analysis of each of the T0 transgenic rice plants at the seedling stage revealed that each promoter deletion conveyed a strong GUS expression in leaves and stems (Figs. 7, 8, 9, Additional file 1: Figs. S13, S14) and a marginal GUS expression in roots as did its promoter (Figs. 7, 8, 9). All of the shortest promoter fragments (199 to 275 bp in length) conveyed strong GUS expression in the leaves and stems of transgenic rice. The only exceptions came from the 464- and 231-bp-long promoter deletions of *PvLhcbp* (i.e., *PvLhcbp*-*1* and -*2*) and the 1210-bp-long deletion of *PvPsbRp* (i.e., *PvPsbRp*-*2*), which conveyed a moderate-to-strong (*PvLhcbp*-*1* and -*2*) and low (*PvPsbRp*-*2*) GUS expression in roots (Figs. 7, 9).

### Green tissue-specific expression of the *PvMYB4* gene in stable transgenic switchgrass

**Fig. 10:**
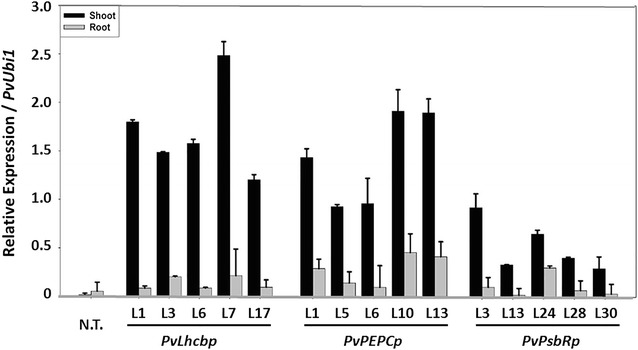
Relative expression of the *PvMYB4* gene in the shoot and root of T0 stable transgenic switchgrass under the control of each of three green tissue-specific promoters measured by real-time RT-PCR. RNA was extracted from at least 10 transgenic lines of each construct at the E5 growth stage with 5 representative lines of each construct being shown. Relative quantification was performed using the standard curve method with *PvUbi1* as the internal control gene. Bars represent the mean values of three independent replicates ± standard errors (vertical bars). N.T., non-transgenic switchgrass

**Fig. 11:**
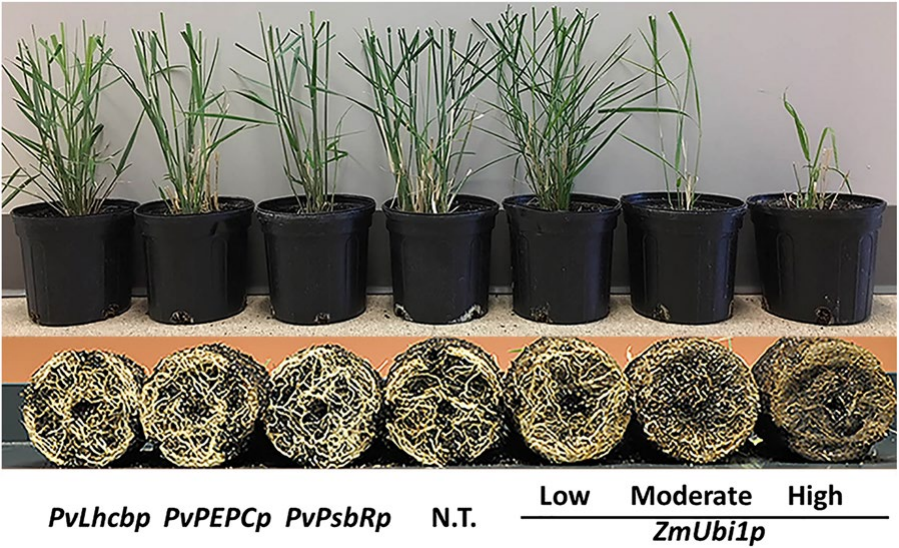
Representative images of the shoots and root systems of pot-grown transgenic switchgrass overexpressing the *PvMYB4* gene under the control of each of the three green tissue-specific promoters. The *PvMYB4* expression levels under the control of the *ZmUbi1* promoter in transgenic switchgrass were low in line 6, moderate in line 8, and high in line 1 [12], and used as the controls. Plants were grown in 4-l pots and images were taken at the R1 growth stage. N.T., non-transgenic switchgrass

In order to examine the application of these green tissue-specific promoters, the three promoters were used to individually drive *PvMYB4* expression in stable transgenic switchgrass. Real-time RT-PCR analysis of 5 randomly selected T0 lines of each promoter showed up to 164-fold overexpression in shoots, but only fourfold overexpression in roots, compared to the expression of the endogenous *PvMYB4* gene in non-transgenic controls (Fig. 10). In contrast to the compromised aboveground growth and the weak and diminished root systems of the transgenic switchgrass ectopically overexpressing the *PvMYB4* gene at high levels under the control of the constitutive *ZmUbi1* promoter [12], green tissue-specific expression of the *PvMYB4* gene in transgenic switchgrass produced normal root systems (Fig. 11). No phenotypic difference was observed in the aboveground tissues between the T0 transgenic switchgrass plants expressing *PvMYB4* under the control of each of the three promoters and the non-transgenic switchgrass (Fig. 11). Thus, aboveground tissue-specific overexpression of *PvMYB4* is an effective strategy to target cell wall phenotype while producing wild-type-like root systems in transgenic switchgrass.

**Fig. 12:**
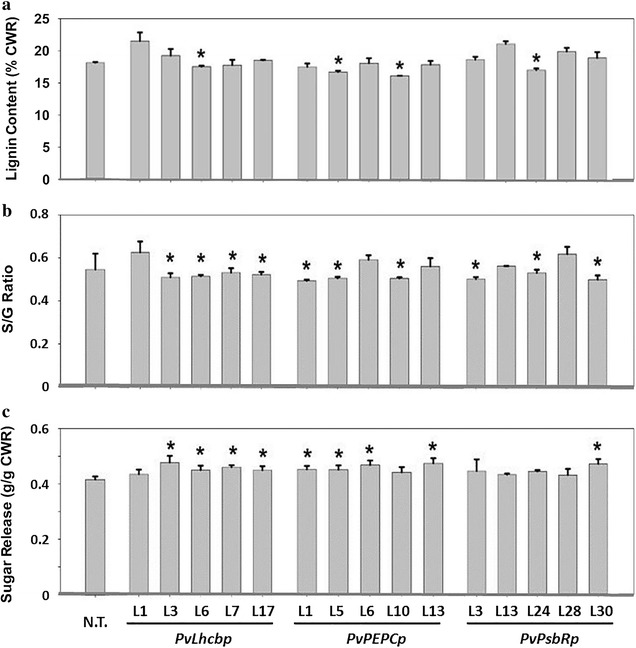
Measurements of total lignin content, S/G lignin monomer ratios, and total cell wall sugar release from the whole tillers of T0 stable transgenic switchgrass under the control of each of three green tissue-specific promoters. The whole tillers were harvested at the E5 growth stage with 5 representative lines of each construct being shown. Total lignin content and S/G lignin monomer ratios were measured using pyrolysis molecular beam mass spectrometry (py-MBMS) of cell wall residues (CWR). Sugar release efficiencies were determined by hot water pretreatment of CWR, followed by enzymatic hydrolysis. Bars represent the mean values of three independent replicates ± standard errors (vertical bars). Bars with asterisk are significantly different from controls at *p *≤ 0.05 as calculated by *t* test. N.T., non-transgenic switchgrass

Total lignin content and S/G lignin monomer ratios were then examined from whole tillers of each of the five randomly selected T0 lines of each promoter using pyrolysis molecular beam mass spectrometry (py-MBMS) of cell wall residues (CWRs). In comparison to the non-transgenic switchgrass, green tissue-specific expression of *PvMYB4* resulted in a significant reduction in total lignin content by 1.9–7.4% in one line of the promoters *PvLhcbp* (line 6) and *PvPsbRp* (line 24), and in two lines of the promoter *PvPEPCp* (lines 5 and 10) (Fig. 12a). Green tissue-specific expression of *PvMYB4* also resulted in a significant reduction in the S/G lignin monomer ratios by 2.9–9.9% in a total of 10 lines of the three promoters, i.e., *PvLhcbp* (4 lines), *PvPEPCp* (3 lines), and *PvPsbRp* (3 lines) (Fig. 12b), including the same four lines with significantly less total lignin content (Fig. 12a). The three lines of both *PvPEPCp* and *PvPsbRp* had comparable reduction in levels of both total lignin content and the S/G lignin monomer ratios, which were greater than the reduction levels in both traits of the four lines of *PvLhcbp*.

Sugar release efficiencies were determined by hot water pretreatment of cell wall residues, followed by enzymatic hydrolysis. All 10 lines including the three different promoter constructs that endowed significant reduction in S/G lignin monomer ratios exhibited significantly higher (9.3–16.2%) release of total sugars compared with the non-transgenic switchgrass (Fig. 12c). Contrasting results in this regard were from 3 lines: *PvPEPCp* (line 10) and *PvPsbRp* (lines 3 and 24), which did not show significant increase in total sugar release than the non-transgenic switchgrass. In addition, lines 6 and 13 of *PvPEPCp*, which did not produce significant reduction in S/G lignin monomer ratios, had significantly increased total sugar release (Fig. 12b, c). Interestingly, the increase in the total sugar release was comparable among all of the above-mentioned 9 lines using the three promoters (Fig. 12c). These 9 lines also exhibited significant increase in xylose release even though only 5 out of the 9 lines had significant increases in glucose release (Additional file 1: Table S1).

## Discussion

The green tissue-specific promoters described here confer high levels of light-inducible gene expression in aboveground tissues, providing an effective means to avoid the side effects caused by transgene expression from constitutive promoters. Here we identified and functionally characterized three switchgrass promoters that were highly green tissue-specific and light-regulated. Their expression patterns were very similar to those of their rice homologs [53–57]. To our best knowledge, these are the first characterized green tissue-specific promoters from switchgrass.

The expression levels of *PvPEPC* detected by real-time RT-PCR were much higher than those of the other two promoters (*PvPsbR* and *PvLhcb*), whereas *PvPEPC* expression level detected by in silico expression analysis was much lower than the other two promoters; *PvPEPC* expression levels detected by the GUS reporter in transgenic rice were comparable to that of the other two promoters. This discrepancy might be a result of inaccuracy of in silico expression analysis. It might also be because *PvPEPCp* provided only a portion of its native promoter strength, partially due to the absence of its 5′UTR. Since *PvPEPCp* comprised the region between the start codons of its own and its nearest upstream gene (*Pavir.J09521.1*) that was reversely orientated, the *PvPEPC* promoter could potentially be a bidirectional promoter. Further experiments are needed to examine the bidirectional functionality of *PvPEPCp*. In addition, the promoter strength of the three green tissue-specific genes in the aboveground tissues of transgenic switchgrass at the heading stage was dramatically lower than that of the *35S* promoter, indicating a developmental variation in strength of the three promoters.

Minimal core promoter regions can be used for parts’ selection for synthetic promoter engineering [58–60]. Most of the 5′-end serial deletions of each promoter provided the same promoter activities as their promoters in transgenic rice, and even the minimal promoter regions tested (199–275 bp in length) were sufficient for providing strong green tissue-specific expression in monocots. These minimal promoters might serve as desirably compact core promoters in monocots, and could be used together with green tissue-specific motifs [50, 51, 61] for synthetic promoter engineering in cereal crops. The findings that the two deletions of *PvLhcbp* (i.e., *PvLhcbp*-*1* and -*2*) and one deletion of *PvPsbRp* (i.e., *PvPsbRp*-*2*) conveyed some GUS expression in roots (Figs. 7, 9) indicate that the promoter regions upstream of *PvLhcbp*-*1* and *PvPsbRp*-*2* might contain sequences inhibiting root expression of both promoters, whereas *PvLhcbp*-*1* and -*2*, and the first 590-bp-long region of *PvPEPCp2* might contain sequences conferring root-preferential expression.

The application of these green tissue-specific promoters in transgenic switchgrass demonstrated that all three promoters restricted *PvMYB4* expression primarily to the aboveground tissues, resulting in maintenance of normal growth of root systems. Surprisingly, we found that the green tissue-specific expression of *PvMYB4* affected the reduction in total lignin content less than the reduction in S/G lignin monomer ratios. This is contrary to what was found for constitutive *PvMYB4* overexpression by the *ZmUbi1* promoter in transgenic switchgrass, which resulted in a greater reduction in total lignin content than in S/G ratio [12, 17]. This discrepancy could reflect the strong activity of the *ZmUbi1* promoter (and hence the *PvMYB4* gene expression) throughout plant development in cell types that do not normally produce secondary cell walls, compared with the green tissue promoters that are only highly active in maturing aboveground tissues that typically form secondary cell walls.

Moreover, the green tissue-specific expression of *PvMYB4* resulted in a smaller increase in total sugar release than observed following constitutive overexpression of *PvMYB4* by the *ZmUbi1* promoter [12]. The green tissue expression of *PvMYB4* also led to more xylose release than glucose release, contrary to constitutive *PvMYB4* overexpression by the *ZmUbi1* promoter [12]. It is worthwhile to point out that we did not find a linear correlation between the *PvMYB4* expression levels and lignin content, S/G ratio, or sugar release. This observation could be attributed to non-linear impacts of changes in the *PvMYB4* transcription factor on the network of downstream genes in the lignin pathway.

The identification and functional characterization of these three green tissue-specific promoters in switchgrass provide novel tools for monocot genetic engineering and synthetic promoter development when aboveground tissues are targeted. Green tissue-specific expression of *PvMYB4* or other transgenes provides a highly effective strategy for crop trait improvement while maintaining unmodified root systems.

## Conclusions

We identified and functionally characterized three green tissue-specific promoters from switchgrass in transgenic rice and switchgrass plants; these promoters could be useful for genetic engineering of monocots when aboveground specificity is preferred. Green tissue-specific expression of *PvMYB4* is an effective strategy for improvement of transgenic biofuel feedstocks.

## Methods

### Switchgrass sequence analysis and in silico expression analysis

The amino acid sequences of the rice *Lhcb*, *PEPC*, and *PsbR* genes were used as the query sequences to BlastP against the switchgrass genomic DNA database (V0.0) on the Phytozome website (www.phytozome.net) in order to obtain their homologous sequences in the switchgrass genome. The International Rice Genome Sequencing Project (IRGSP) gene IDs of the three rice *Lhcb* genes *OsLhcb1*-*1, OsLhcb1*-*2,* and *OsLhcb2*-*1* were Os09g17740 [54, 55, 57], Os1g41710 [54], and Os03g39610 [55], respectively. The IRGSP gene IDs of the five plant-type rice *PEPC* genes *Osppc1*, *2a*, *2b*, *3,* and *4* were Os02g0244700, Os08g0366000, Os09g0315700, Os01g0758300, and Os01g0208700, respectively [56]. The IRGSP gene IDs of the three rice *PsbR* genes *OsPsbR1, 2*, and *3* were Os07g05360, Os07g05365, and Os08g10020, respectively [53]. The deduced amino acid sequences of the switchgrass homologs of these rice genes were aligned together with their respective query protein sequences using ClustalX 2.0 [62] (Additional file 1: Fig. S1). The promoter sequences (just upstream the start codon), genomic DNA sequences, and cDNA (including 5′ and 3′UTR) sequences of each switchgrass homolog were obtained from Phytozome. The unitranscript entry of each switchgrass homolog was obtained from the Noble Foundation switchgrass gene expression atlas PviUT V1.2 (http://switchgrassgenomics.noble.org; [63]) using each switchgrass cDNA sequence as the query. The switchgrass gene expression profiles were then obtained from the Noble Foundation switchgrass gene expression atlas (https://switchgrassgenomics.noble.org/download_seq.php; [63]).

### Characterization of target gene expression in non-transgenic switchgrass

Switchgrass cv. ‘Alamo’ was used for tissue-specific RNA and genomic DNA extraction. Switchgrass plants were grown in the greenhouse at 28 °C under 16-h day/8-h night photoperiods (390 μE/m^2^/s). The dark/light experiments were conducted by germinating cv. ‘Alamo’ seeds at 28 °C and growing seedlings under 16-h day/8-h night photoperiods (390 μE/m^2^/s) or without light for three weeks until RNA extraction.

### Switchgrass genomic DNA extraction, PCR amplification, and vector construction

Switchgrass genomic DNA was extracted from the leaf blade at the R1 growth stage [64] using a CTAB method [65]. The genomic DNA was used as the template for PCR amplification of the promoter regions of each green tissue-specific gene using sequence-specific primers (Table 1). The PCR product of each gene was gel purified and cloned into the pCR™8/GW/TOPO^®^ vector (Invitrogen™, Carlsbad, CA, USA) for sequencing confirmation. Each promoter was subcloned upstream of the *uidA* (*GUS*) gene in the pMDC162 vector with the help of the Gateway^®^ LR Clonase™ II enzyme (Invitrogen™, Carlsbad, CA, USA) for sequence confirmation and then stable rice transformation (see below).

**Table 1:** Primer sequences used in this study

Gene	Primer	Primer Sequence (5′ > 3′)	Application
*PvLhcb*	PPvLhcb1-2F	CCCCGACCGATGCATCTACA	Promoter cloning
	PPvLhcb1-2R*	TGAGCCGAAGGAGGGTTGCT	Promoter cloning
	5Lhcb1-2-1F	CCTGTCACACACACAAGAGATGGC	Serial deletion
	5Lhcb1-2-2F	GTGAGAATATCTGGCGGCGAGC	Serial deletion
	Lhcb1qPCRF2	CTCGCCGACCACCTCACCGAT	qRT-PCR
	Lhcb1qPCRR1	CGGAAGACAATGAAGTTGCAGA	qRT-PCR
*PvPEPC*	PPvPEPCF	CACCGGCACTCTATGCTTAC	Promoter cloning
	PPvPEPCR*	CTAGCTAGCAGCTGCAGTTG	Promoter cloning
	5PEPC-1F	CGAGGAGCAGAAGAAGTCAC	Serial deletion
	5PEPC-2F	GCCACAGATTGACATGAAGTATCAC	Serial deletion
	5PEPC-3F	GCTCTAGTGTGAACGAAGTTCC	Serial deletion
	5PEPC-4F	CAGTAGCAGTACGGCAAGTC	Serial deletion
	PEPCqPCRF1	CTCATCCTCACCATGAAGGGT	qRT-PCR
	PEPCqPCRR1	TGCAGTTGAGGAGCGGAGCGC	qRT-PCR
*PvPsbR*	PPvPsbRF	GTTGGGCTGGTCTTGATCGT	Promoter cloning
	PPvPsbRR*	TTGCTGCTGCTTCGATTGCC	Promoter cloning
	PsbR-1F	ACGCACGGAATCACCTAGCAC	Serial deletion
	PsbR-2F	CAGCCCTGTATGCATGAAGTCC	Serial deletion
	PsbR-3F	GTTGGATGGCCTATCTTGTCCGG	Serial deletion
	PsbR-4F	GAGTCGATGCAGCATCCTCGATC	Serial deletion
	PsbR-5F	TCCCCAGAGGATAACGTTGCAG	Serial deletion
	PsbRqPCRF1	CCTTGTTTACAACACCAGCGCT	qRT-PCR
	PsbRqPCRR2	CCCAAGCTGATCTTACATG	qRT-PCR
*PvUbi1*	Ubi1-F	CAGCGAGGGCTCAATAATTCCA	qRT-PCR
	Ubi1-R	TCTGGCGGACTACAATATCCA	qRT-PCR
*PvMYB4* ^a^	MYB4-T-F	TCGGCATGCTCCTCGACTTC	qRT-PCR
	MYB4-T-R	ATCATAGGCGTCTCGCATATCT	qRT-PCR
*PvMYB4* ^b^	MYB4-E-F	AGGCCTCGAGATGAAGTGAAAC	qRT-PCR
	MYB4-E-R	AGCCCAACAAACAAACGAAATT	qRT-PCR

qRT-PCR, real-time RT-PCR

*Used as the reverse primers for cloning of the serial deletions and the promoter of each promoter

^a^Primers for the transgene

^b^Primers for the gene specific (endogenous)

Similarly, each 5′-end serial deletion of each green tissue-specific promoter was PCR amplified and cloned into pCR™8/GW/TOPO^®^ for sequencing confirmation, and then subcloned into pMDC162 for stable rice transformation. The switchgrass *Ubiquitin 2* (i.e., *PvUbi2*), maize *Ubiquitin 1* (i.e., *ZmUbi1*), and CaMV *35S* promoters were also cloned into pMDC162, which was used as the positive control vector.

### Switchgrass RNA extraction and real-time RT-PCR

Total RNA was extracted from leaf blade, leaf sheath, inflorescence, internode, node, root, and seeds of switchgrass (cv. Alamo) or shoots and roots of transgenic switchgrass expressing *PvMYB4* under the control of each green tissue-specific promoter at the E5 stage using Tri-Reagent (Molecular Research Center, Cincinnati, OH, USA). RNA quality was analyzed with an Agilent 2100 Bioanalyzer (Agilent Technologies, Santa Clara, CA, USA). One µg of RNA from each tissue was treated with DNase I (Invitrogen™, Carlsbad, CA, USA) and then used for reverse transcription with the High-Capacity cDNA Reverse Transcription kit (Applied Biosystems, Foster City, CA, USA).

The relative expression of each endogenous green tissue-specific gene in each switchgrass tissue or the *PvMYB4* transgene in the shoots or roots of transgenic switchgrass was quantified by real-time quantitative reverse transcription PCR (real-time RT-PCR) using sequence-specific primers (Table 1). A switchgrass *Ubiquitin* gene (i.e., *PvUbi1*) was used as the internal control (Table 1) [29]. The real-time RT-PCR reactions were conducted using the Power SYBR Green PCR master mix (Applied Biosystems, Foster City, CA, USA) on a 7900HT Fast Real-Time PCR System (Applied Biosystems, Foster City, CA, USA). The standard curve method was used for relative expression analysis normalized by *PvUbi1* [29].

### Rice transformation

Seeds of japonica rice (*O. sativa* L.) cv. TP309 were provided by the USDA National Plant Germplasm System. All of the promoters and serial deletions driving *GUS* expression were introduced into cv. ‘TP309 by *Agrobacterium*-mediated transformation [66]. Transgenic rice plants were grown in growth chambers at 27 °C under 12-h photoperiods for 2 weeks before being transferred to a greenhouse and grown at 25–29 °C under 12-h photoperiods.

### Histochemical GUS analysis in transgenic rice

Histochemical GUS assay was performed according to the published protocol [67]. The leaf, stem, and root of each individual plant were incubated in GUS staining solution (200 mM potassium phosphate at pH 7.0, 0.1% Triton X-100, 1 mg/ml X-Gluc, 10% DMSO) at 37 °C for 6–10 h. After staining, the samples were bleached with 70% (v/v) ethanol, and images were taken under a dissecting microscope (Fisher Scientific™ Stereomaster™ Track Pole, Pittsburgh, PA, USA) using a digital camera (Infinity X-32, Lumenera Corporation, Ottawa, ON, USA). For each construct, at least ten independent transgenic lines were subjected to histochemical GUS assays.

### Fluorometric GUS assay in transgenic rice

Quantitative fluorometric assay for GUS activities was conducted according to the published protocol [67]. Total protein concentration was quantified by the Bradford assay [68]. A fixed excitation (365 nm)–emission (460 nm) wavelength fluorometer was used to determine the relative fluorescence units of each sample for three times at intervals of 10 min. Calibration was performed by reading 1000 units for 100 pmol of 4-methylumbelliferone (4-MU) using a 100 nM MU solution. Results were expressed as pmol 4-MU produced (mg protein)/min.

### Switchgrass transformation

Each of the three green tissue-specific promoters was individually cloned into the 5′-end of the *PvMYB4* gene [17] with the octopine synthase terminator (OCST) being the terminator. Each cassette (individual promoter-*PvMYB4*-OCST) was subcloned into pMDC99 for sequencing confirmation and stable switchgrass cv. ‘Alamo’ transformation [31].

### Analysis of lignin content and composition and cell wall sugar release in transgenic switchgrass

Lignin content, composition, and sugar release from cell wall residues were measured as previously described [69]. Specifically, 300 mg shoot samples were pyrolyzed at 500 °C in 80-µl stainless steel cups using an Extrel single-quadrupole molecular beam mass spectrometer. Lignin content was determined from the relative intensities of the peaks corresponding to the lignin monomers, while S/G ratio was calculated by dividing the sum of the intensities of the syringyl peaks by the sum of the intensities of the guaiacyl peaks.

Sugar release was determined by high-throughput pretreatment and enzymatic hydrolysis [12, 70]. Amylases were used to remove soluble extractives and starch from the biomass samples, followed by ethanol extraction in a Soxhlet extractor [71]. The resulting cell wall residues were loaded into custom-made 96-well metal plates in triplicate. Samples containing ~ 1.7% solids in water (w/w) were pretreated with condensing steam at 180 °C for 17.5 min. Then, enzymatic hydrolysis was conducted by incubation at 50 °C for 70 h with Ctec2 enzyme cocktail (Novozymes North America, Franklinton, NC) at 70 mg protein/g biomass. Glucose and xylose released into the liquid were quantified by colorimetric assays (Megazyme Intl., Bray, Ireland).

### Statistical analysis

Analysis of variance (ANOVA) was performed for statistical analyses (*p* < 0.05) (SAS 9.2 for Windows; SAS Institute, Cary, NC).

## Additional file

**Additional file 1: Table S1.** Sugars (g/g CWR) released by enzymatic hydrolysis from the transgenic switchgrass lines expressing *PvMYB4* under the control of each of the three green tissue-specific promoters. **Figure S1.** Comparison of the deduced amino acid sequences of the rice *Lhcb* genes and their homologs in switchgrass. **Figure S2.** Comparison of the deduced amino acid sequences of the rice *PEPC* gene and its homologs in switchgrass. **Figure S3.** Comparison of the deduced amino acid sequences of the rice *PsbR* genes and their homologs in switchgrass. **Figure S4.** The gene structures of the three rice *Lhcb* genes (i.e., *OsLhcb1*-*1, OsLhcb1*-*2*, and *OsLhcb2*-*1*, whose International Rice Genome Sequencing Project (IRGSP) gene IDs are Os09g17740 [54, 55, 57], Os1g41710 [54], and Os03g39610 [55], respectively) and their switchgrass homologs with the highest amino acid sequence similarities. **Figure S5.** The gene structures of the five plant-type rice *PEPC* genes (i.e., *Osppc1*, *2a*, *2b*, *3*, and *4*, whose International Rice Genome Sequencing Project (IRGSP) gene IDs are Os02g0244700, Os08g0366000, Os09g0315700, Os01g0758300, and Os01g0208700, respectively [56]) and their switchgrass homologs with the highest amino acid sequence similarities. **Figure S6.** The gene structures of the three rice *PsbR* genes (i.e., *OsPsbR1, 2* and *3*, whose International Rice Genome Sequencing Project (IRGSP) gene IDs are Os07g05360, Os07g05365, and Os08g10020, respectively [53]) and their switchgrass homologs with the highest amino acid sequence similarities. **Figure S7.** The in silico expression profiles of the unitranscript entries of the potential switchgrass homologs of *OsLhcb1*-*1*, *OsLhcb1*-*2*, and *OsLhcb2*-*1*, whose International Rice Genome Sequencing Project (IRGSP) gene IDs are Os09g17740 [54, 55, 57], Os1g41710 [54], and Os03g39610 [55], respectively, in different tissues of non-transformed switchgrass. **Figure S8.** The in silico expression profiles of the unitranscript entries of the potential switchgrass homologs of *Osppc1*, *2a*, *2b*, *3*, and *4*, whose International Rice Genome Sequencing Project (IRGSP) gene IDs are Os02g0244700, Os08g0366000, Os09g0315700, Os01g0758300, and Os01g0208700, respectively [56], in different tissues of non-transformed switchgrass. **Figure S9.** The in silico expression profiles of the unitranscript entries of the potential switchgrass homologs of *OsPsbR1, 2*, and *3*, whose International Rice Genome Sequencing Project (IRGSP) gene IDs are Os07g05360, Os07g05365, and Os08g10020, respectively [53], in different tissues of non-transformed switchgrass. **Figure S10.** The 764-bp-long promoter sequence of *PvLhcb* (i.e., *Pavirv00047797m*) used in the present study. **Figure S11.** The 1878-bp-long promoter sequence of *PvPEPC* (i.e., *Pavirv00033161m*) used in the present study. **Figure S12.** The 2009-bp-long promoter sequence of *PvPsbR* (i.e., *Pavirv00009702m*) used in the present study. **Figure S13.** Quantitative fluorometric GUS analysis of leaf blade, leaf sheath, stem, and panicles of T0 stable transgenic rice containing each serial deletion of the *PvLhcb* promoter at the heading stage. **Figure S14.** Quantitative fluorometric GUS analysis of leaf blade, leaf sheath, stem, and panicles of T0 stable transgenic rice containing each serial deletion of the *PvPEPC* promoter at the heading stage.

## Abbreviations

*Lhcb*: photosynthesis-related light-harvesting complex II chlorophyll-a/b binding gene
*PEPC*: phosphoenolpyruvate carboxylase
*PsbR*: the subunit R of the photosystem II 10 kDa polypeptide
