# Supplementary material for: Switchgrass (Panicum virgatum L.) promoters for green tissue-specific expression of the MYB4 transcription factor for reduced-recalcitrance transgenic switchgrass
Source: Biotechnol Biofuels. 2018 Apr 24;11:122. doi: 10.1186/s13068-018-1119-7 (PMC5914048; doi:10.1186/s13068-018-1119-7)

**Additional file 1**

**Additional supporting information may be found in the online version of this article:**

**Table S1. Sugars (g/g CWR) released by enzymatic hydrolysis from the transgenic switchgrass lines expressing *PvMYB4* under the control of each of the three green tissue-specific promoters.**

| **Promoter** | **Line** | **Glycose release** | **Xylose release** | **Total release** |
| --- | --- | --- | --- | --- |
| Non-transgenic |  | 0.246 ± 0.005 | 0.172 ± 0.07 | 0.418 ± 0.011 |
| *PvLhcbp* | L1 | 0.240 ± 0.005 | **0.203 ± 0.012*** | 0.443 ± 0.017 |
|  | L3 | 0.260 ± 0.023 | **0.226 ± 0.003*** | **0.486 ± 0.024*** |
|  | L6 | 0.258 ± 0.006 | **0.200 ± 0.016*** | **0.458 ± 0.016*** |
|  | L7 | **0.267 ± 0.007*** | **0.202 ± 0.002*** | **0.469 ± 0.007*** |
|  | L17 | 0.253 ± 0.008 | **0.205 ± 0.007*** | **0.458 ± 0.014*** |
| *PvPEPCp* | L1 | **0.263 ± 0.007*** | **0.196 ± 0.005*** | **0.459 ± 0.012*** |
|  | L5 | **0.262 ± 0.011*** | **0.195 ± 0.005*** | **0.457 ± 0.016*** |
|  | L6 | **0.266 ± 0.009*** | **0.209 ± 0.008*** | **0.474 ± 0.017*** |
|  | L10 | **0.267 ± 0.011*** | 0.181 ± 0.008 | 0.448 ± 0.019 |
|  | L13 | **0.265 ± 0.013*** | **0.216 ± 0.008*** | **0.481 ± 0.019*** |
| *PvPsbRp* | L3 | 0.244 ± 0.029 | **0.202 ± 0.013*** | 0.446 ± 0.042 |
|  | L13 | 0.242 ± 0.002 | 0.191 ± 0.003 | 0.433 ± 0.004 |
|  | L24 | 0.260 ± 0.007 | 0.184 ± 0.002 | 0.445 ± 0.005 |
|  | L28 | 0.244 ± 0.010 | 0.187 ± 0.012 | 0.431 ± 0.022 |
|  | L30 | 0.263 ± 0.008 | **0.209 ± 0.009*** | **0.471 ± 0.018*** |

Three biological replicates (individual plants) from each transgenic line and the non-transgenic switchgrass were used in the analysis. Values represent the average of the biological replicates ± standard deviations, and asterisks (and bold numerical numbers) indicate the significant differences in comparison to the non-transgenic switchgrass as determined by a *t*-test (*P* < 0.05). CWR, cell wall residues.

**Figure S1.** **Comparison of the deduced amino acid sequences of the rice *Lhcb* genes and their homologs in switchgrass.**

The homologous sequences of the 3 rice *Lhcb* genes (i.e., *OsLhcb1-1, OsLhcb1-2* and *OsLhcb2-1*, whose International Rice Genome Sequencing Project (IRGSP) gene IDs are Os09g17740 [54,55,57], Os1g41710 [54] and Os03g39610 [55], respectively) in the switchgrass genome were obtained by using the amino acid sequence of *OsLhcb1-1* as the query sequence to BlastP against the switchgrass genomic DNA sequence database in Phytozome (<http://www.phytozome.net/search.php>). The promoter region of *Pavirv00047797m* was used in the present study. Dots represent the identical amino acid residues, dashes represent gaps, and the numbers indicate the positions of the amino acid residues.


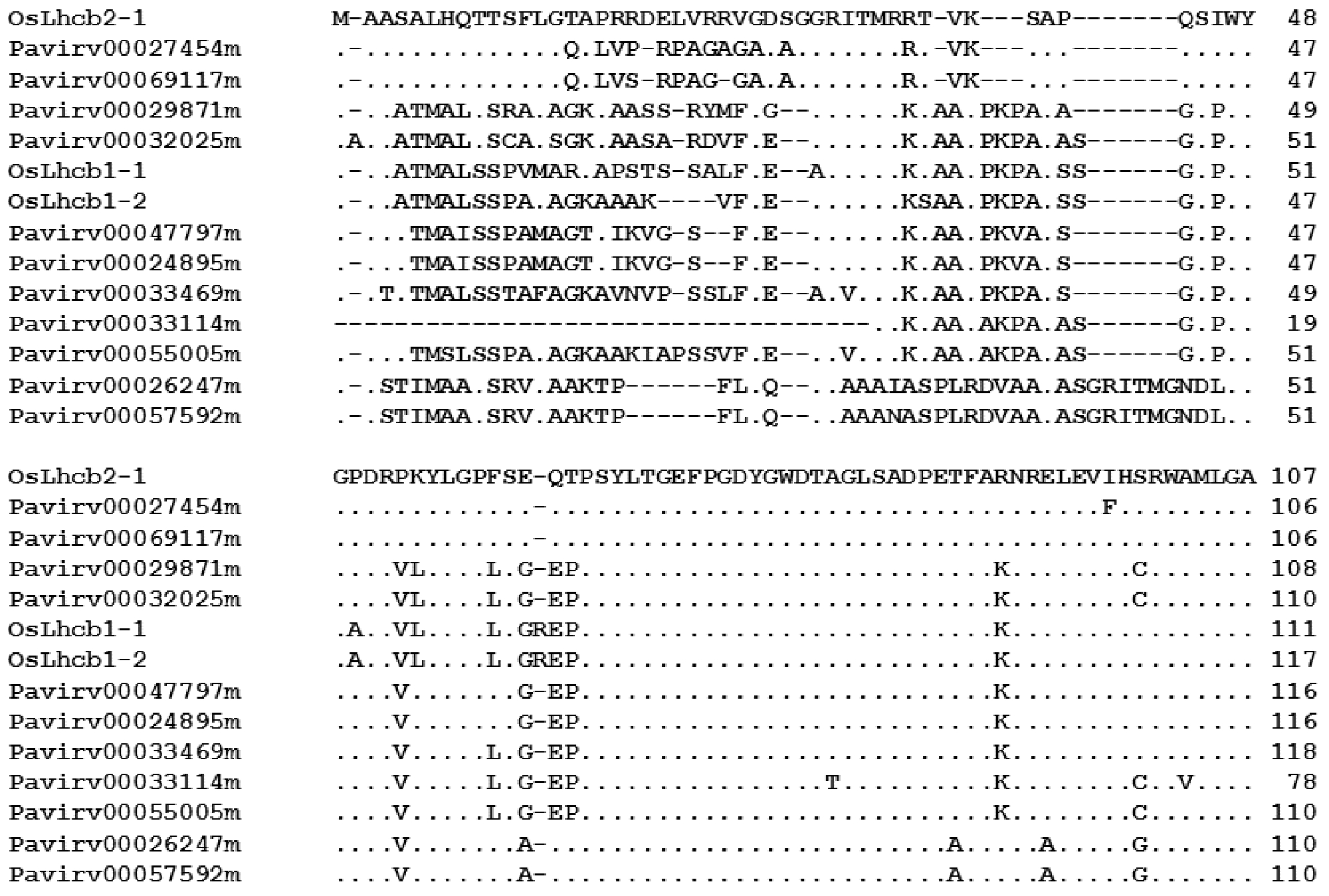


**Figure S1. (Cont.)**


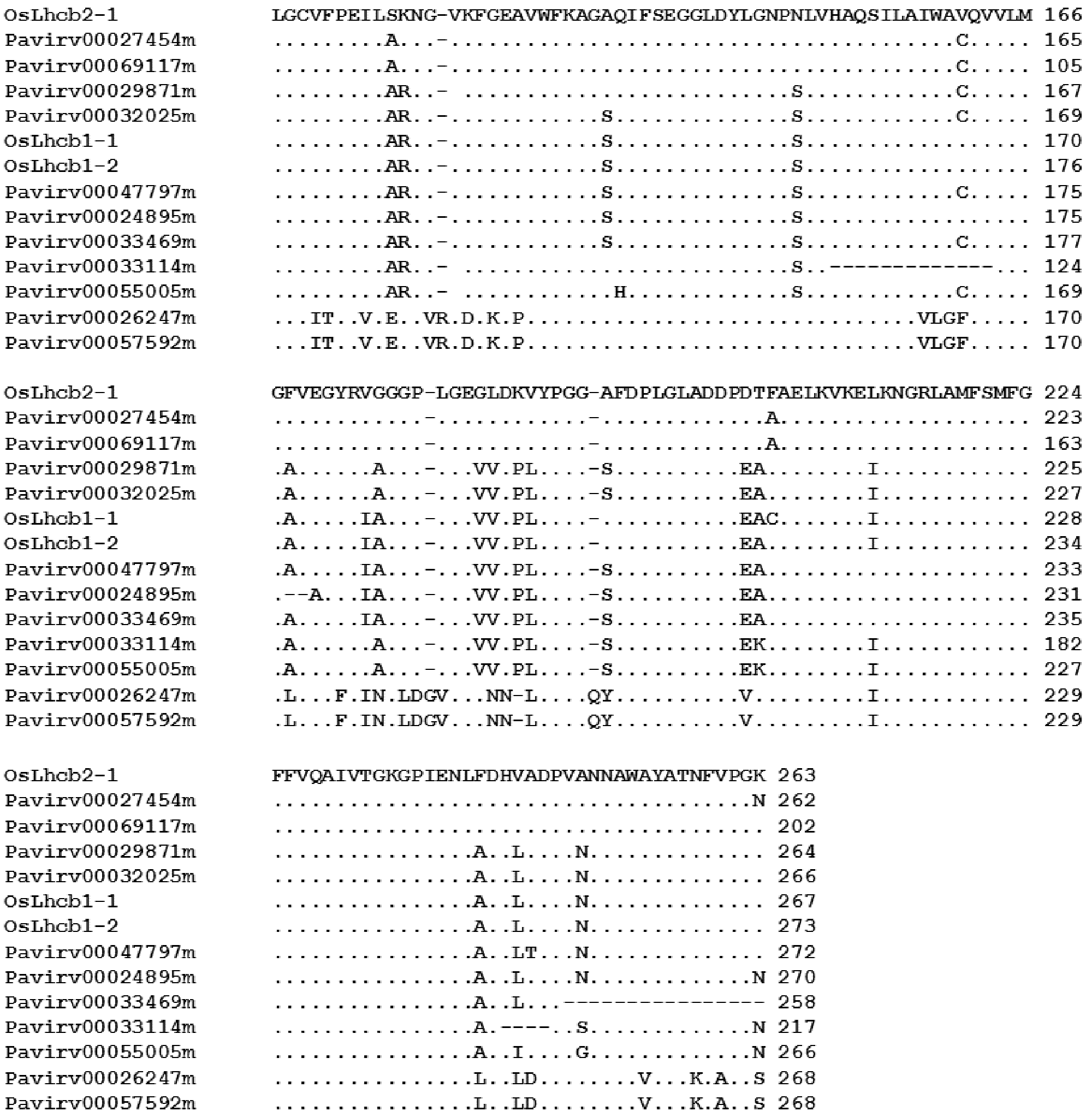


**Figure S2.** **Comparison of the deduced amino acid sequences of the rice *PEPC* gene and its homologs in switchgrass.**

The homologous sequences of the 5 plant-type rice *PEPC* genes (i.e., *Osppc1*, *2a*, *2b*, *3* and *4*, whose International Rice Genome Sequencing Project (IRGSP) gene IDs are Os02g0244700, Os08g0366000, Os09g0315700, Os01g0758300, and Os01g0208700, respectively [56]) in the switchgrass genome were obtained by using the amino acid sequence of *Osppc1* as the query sequence to BlastP against the switchgrass genomic DNA sequence database in Phytozome (<http://www.phytozome.net/search.php>). The promoter region of *Pavirv00033161m* was used in the present study. Dots represent the identical amino acid residues, dashes represent gaps, and the numbers indicate the positions of the amino acid residues.


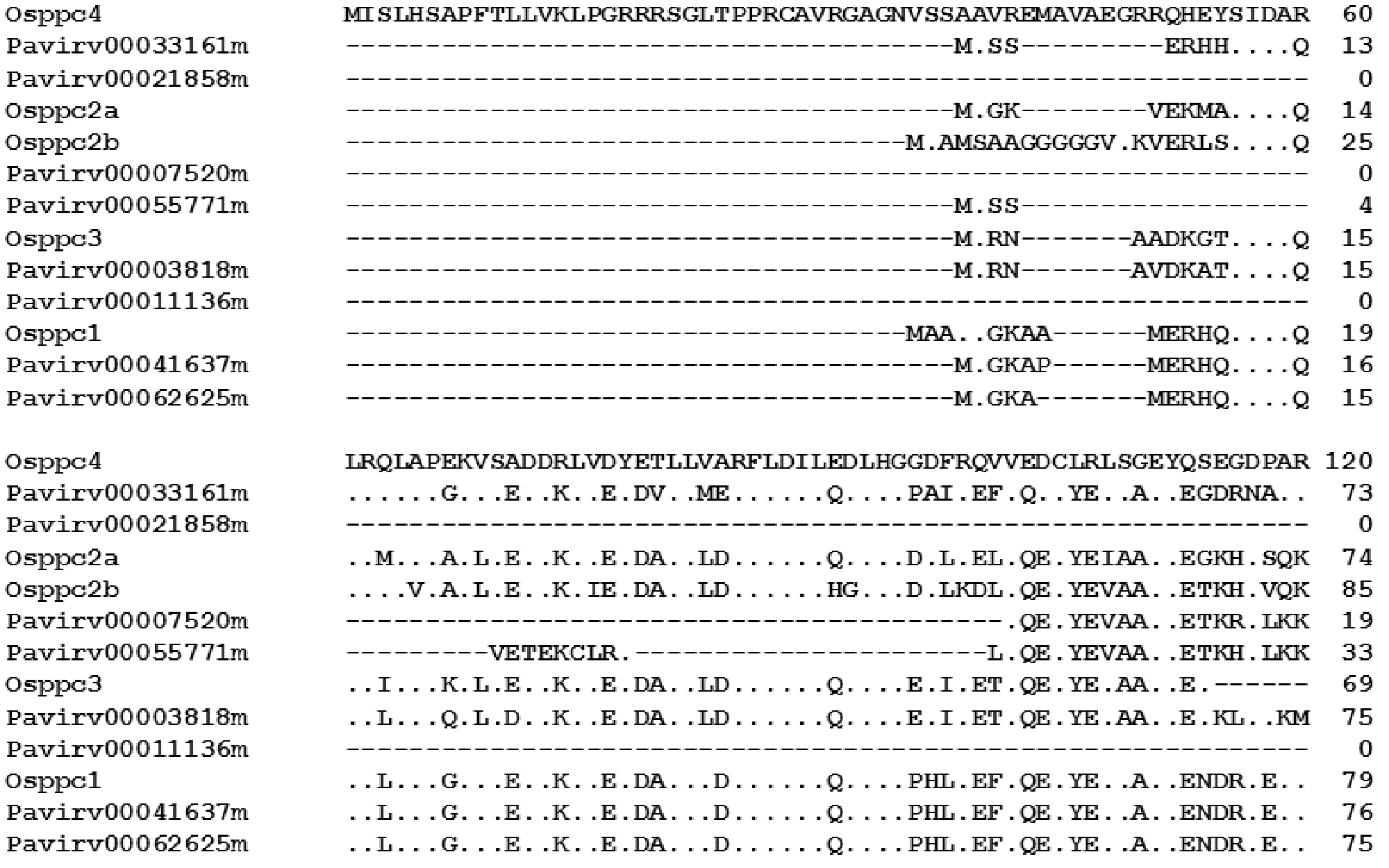


**Figure S2. (Cont.)**


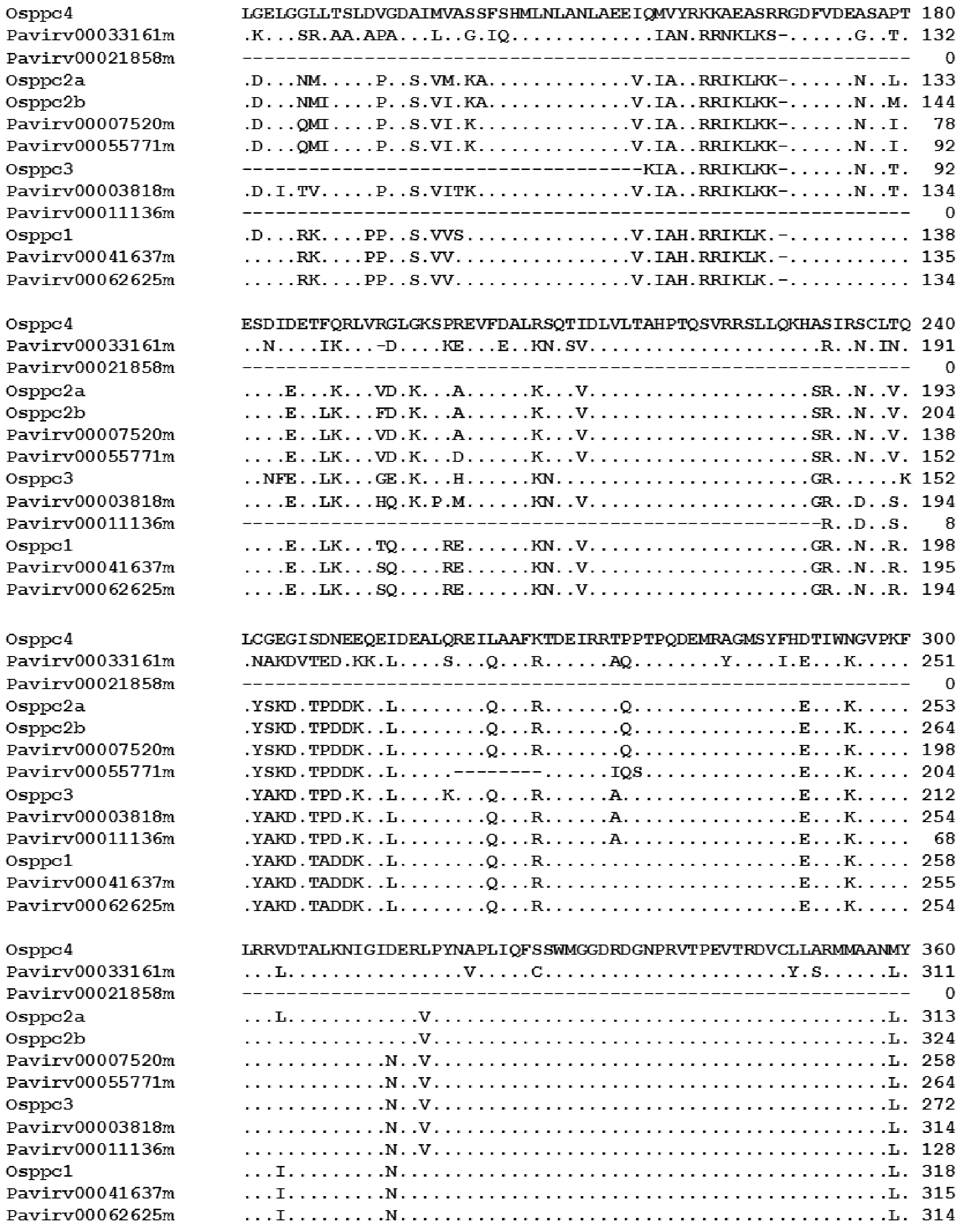


**Figure S2. (Cont.)**


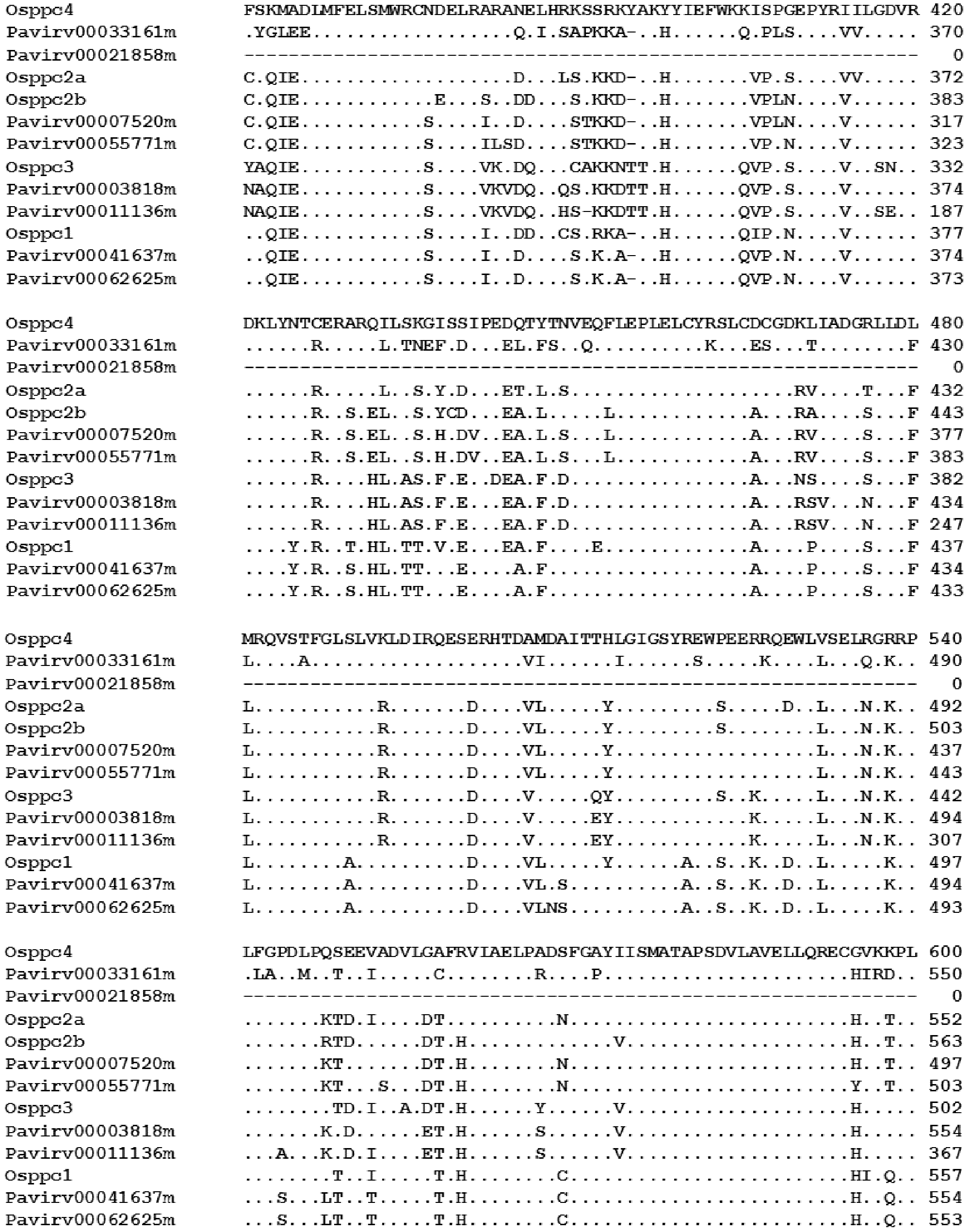


**Figure S2. (Cont.)**


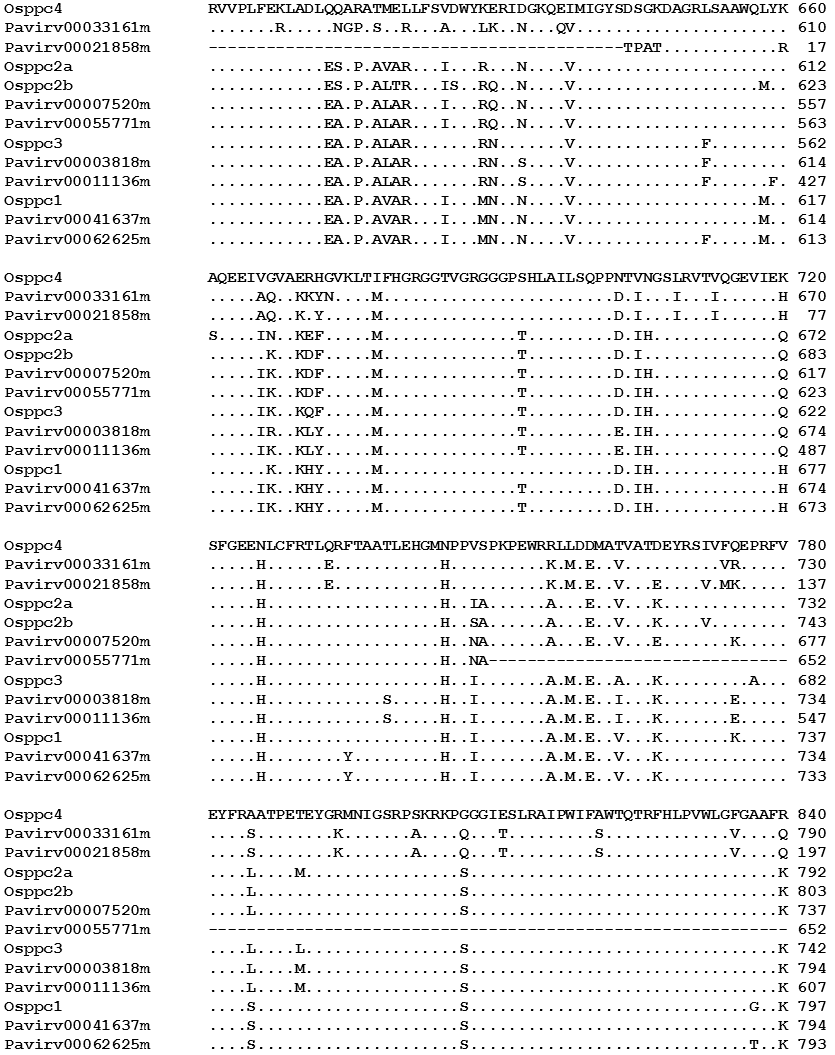


**Figure S2. (Cont.)**


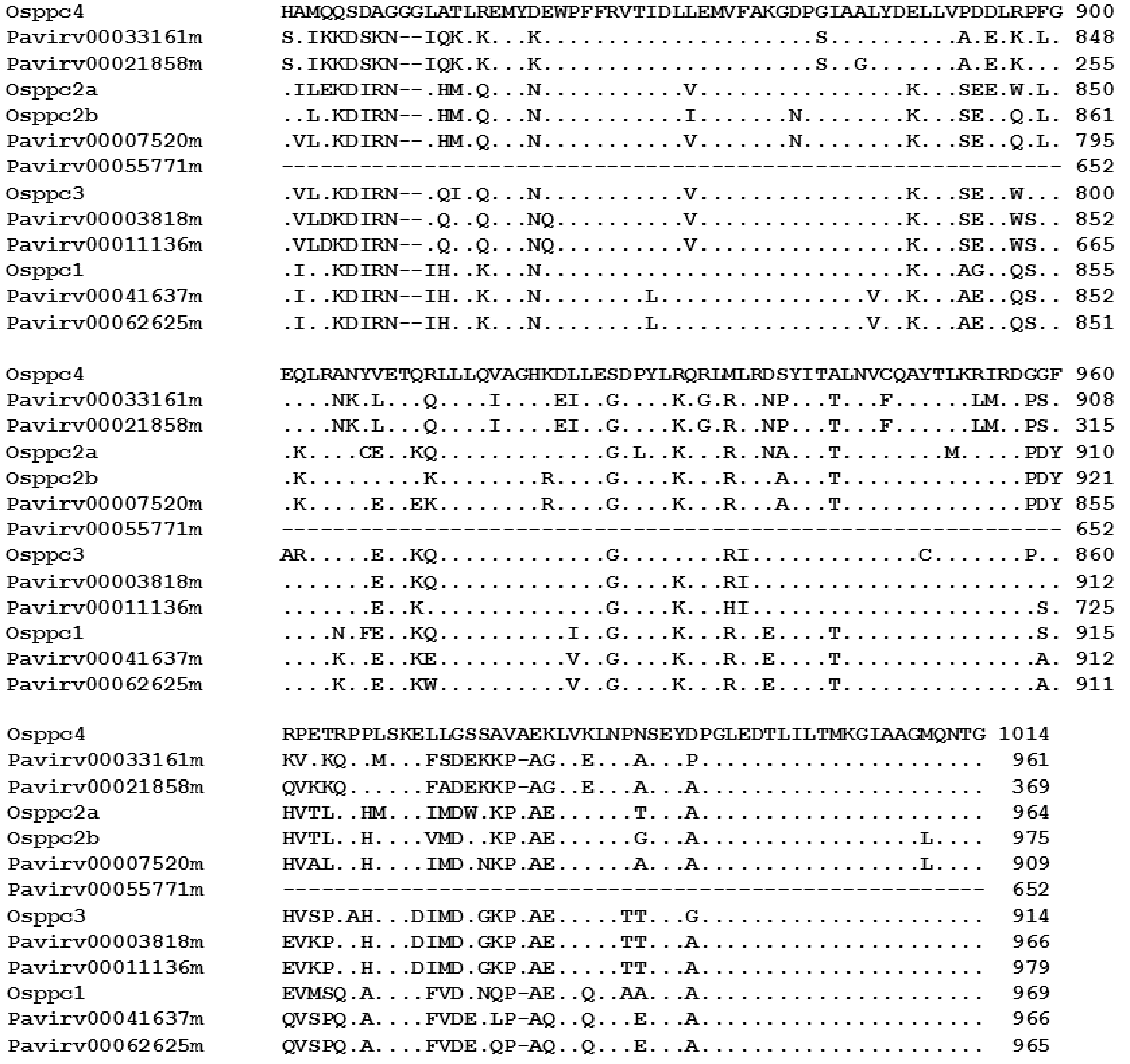


**Figure S3.** **Comparison of the deduced amino acid sequences of the rice *PsbR* genes and their homologs in switchgrass.**

The homologous sequences of the three rice *PsbR* genes (i.e., *OsPsbR1, 2* and *3*, whose International Rice Genome Sequencing Project (IRGSP) gene IDs are Os07g05360, Os07g05365, and Os08g10020, respectively [53]) in the switchgrass genome were obtained by using the amino acid sequence of *OsPsbR1* as the query sequence to BlastP against the switchgrass genomic DNA sequence database in Phytozome (<http://www.phytozome.net/search.php>). The promoter region of *Pavirv00009702m* was used in the present study. Dots represent the identical amino acid residues, dashes represent gaps, and the numbers indicate the positions of the amino acid residues.


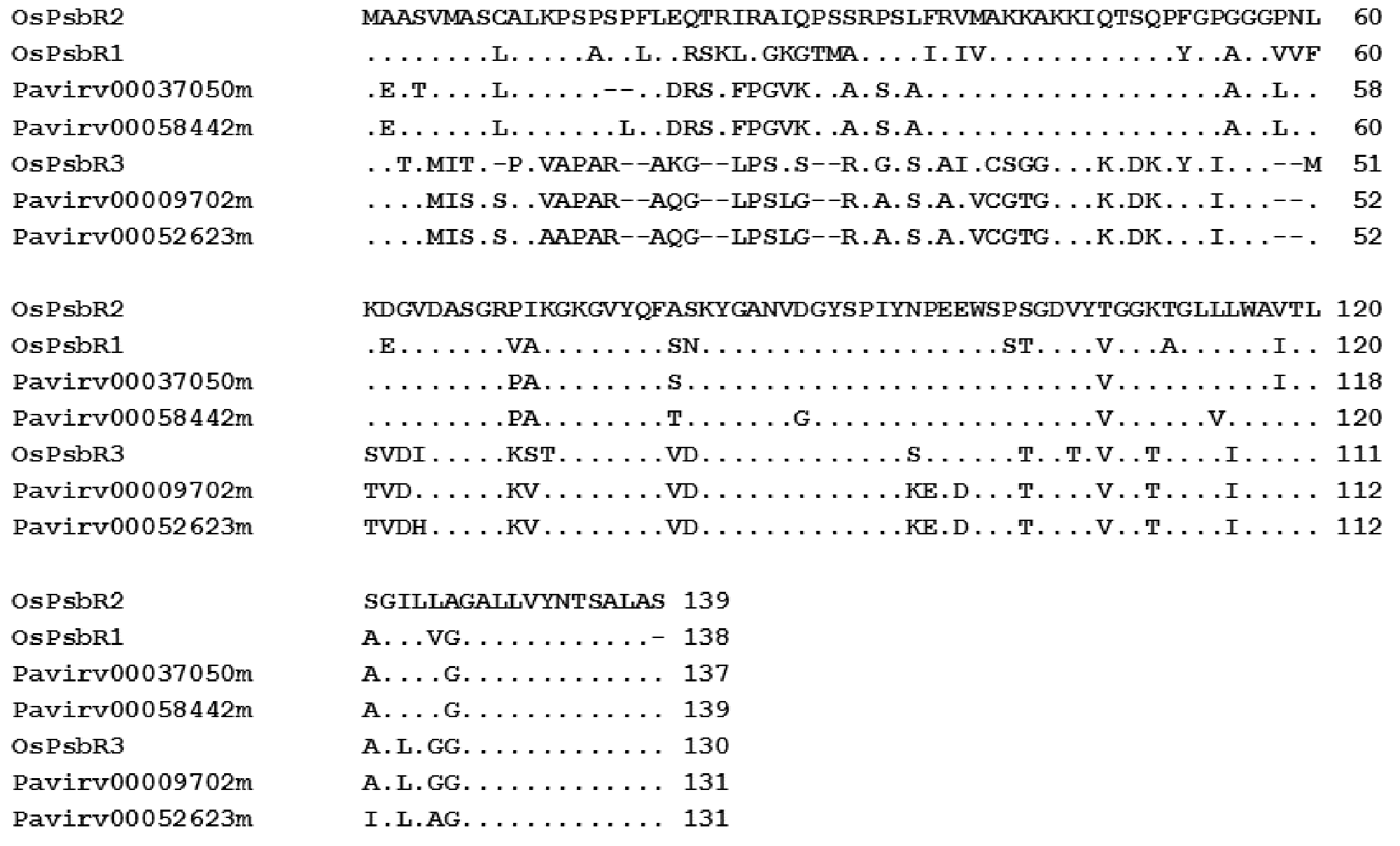


**Figure S4. The gene structures of the three rice *Lhcb* genes (i.e., *OsLhcb1-1, OsLhcb1-2* and *OsLhcb2-1*, whose International Rice Genome Sequencing Project (IRGSP) gene IDs are Os09g17740 [54,55,57], Os1g41710 [54] and Os03g39610 [55], respectively)** **and their switchgrass homologs with the highest amino acid sequence similarities.** The promoter region of *Pavirv00009702m* was used in the present study. Horizontal and vertical lines represent exons and introns, respectively, with exon length (aa; amino acids) being indicated under each exon.

**
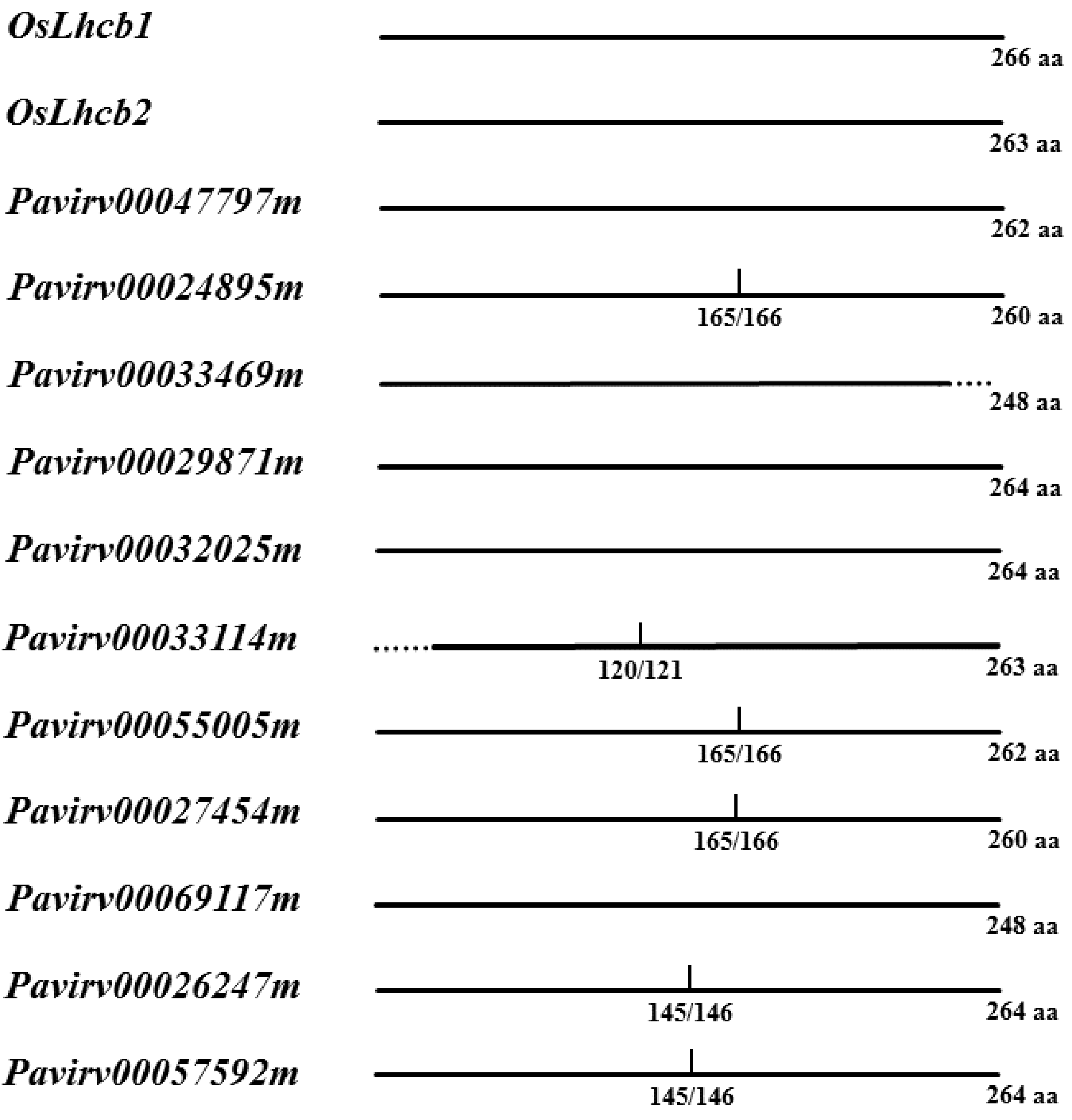
**

**Figure S5. The gene structures of the five plant-type rice *PEPC* genes (i.e., *Osppc1*, *2a*, *2b*, *3* and *4*, whose International Rice Genome Sequencing Project (IRGSP) gene IDs are Os02g0244700, Os08g0366000, Os09g0315700, Os01g0758300, and Os01g0208700, respectively [56]) and their switchgrass homologs with the highest amino acid sequence similarities.** The promoter region of *Pavirv00033161m* was used in the present study. Horizontal and vertical lines represent exons and introns, respectively, with exon length (aa; amino acids) being indicated under each exon.


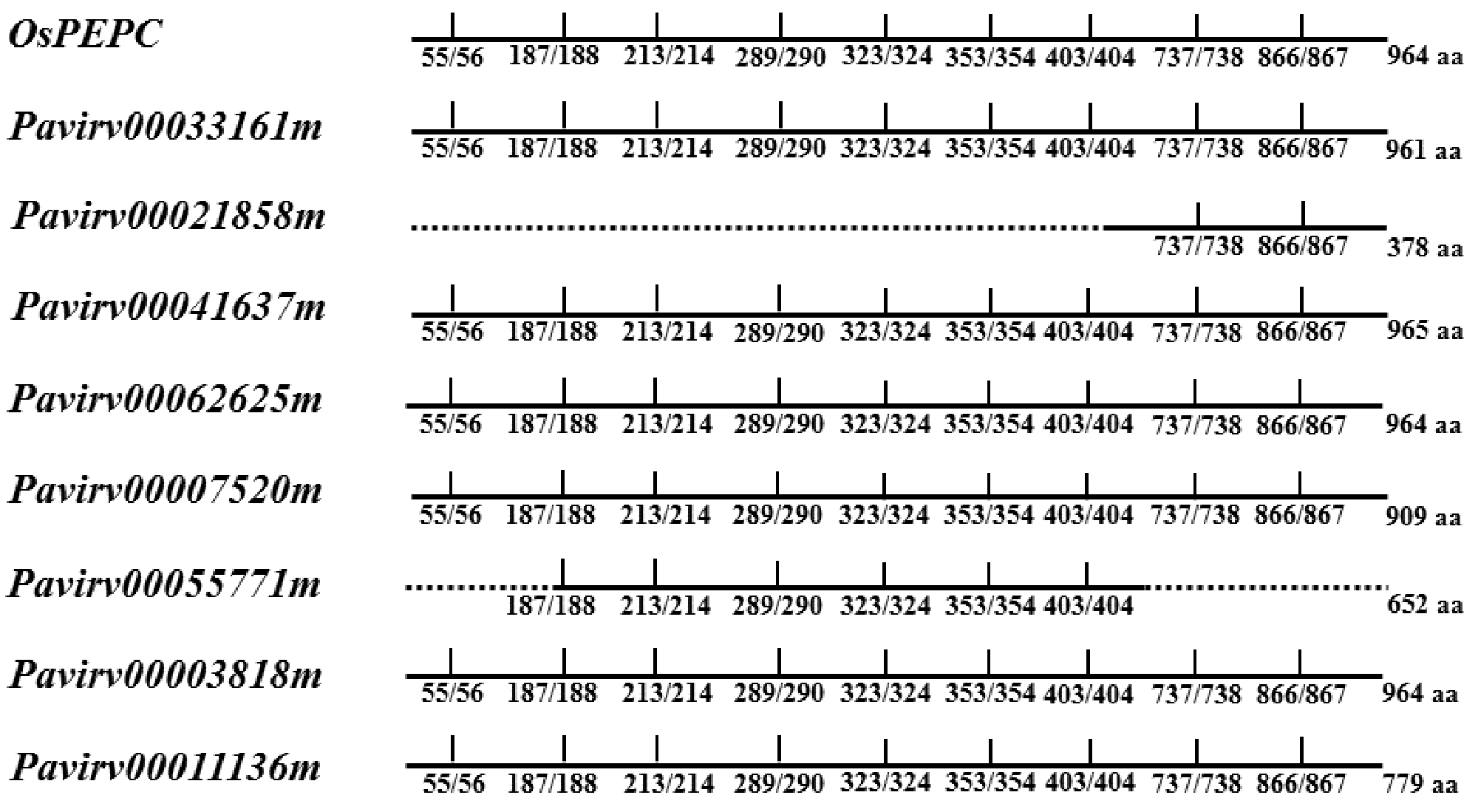


**Figure S6. The gene structures of the three rice *PsbR* genes (i.e., *OsPsbR1, 2* and *3*, whose International Rice Genome Sequencing Project (IRGSP) gene IDs are Os07g05360, Os07g05365, and Os08g10020, respectively [53]) and their switchgrass homologs with the highest amino acid sequence similarities.** The promoter region of *Pavirv00009702m* was used in the present study. Horizontal and vertical lines represent exons and introns, respectively, with exon length (aa; amino acids) being indicated under each exon.


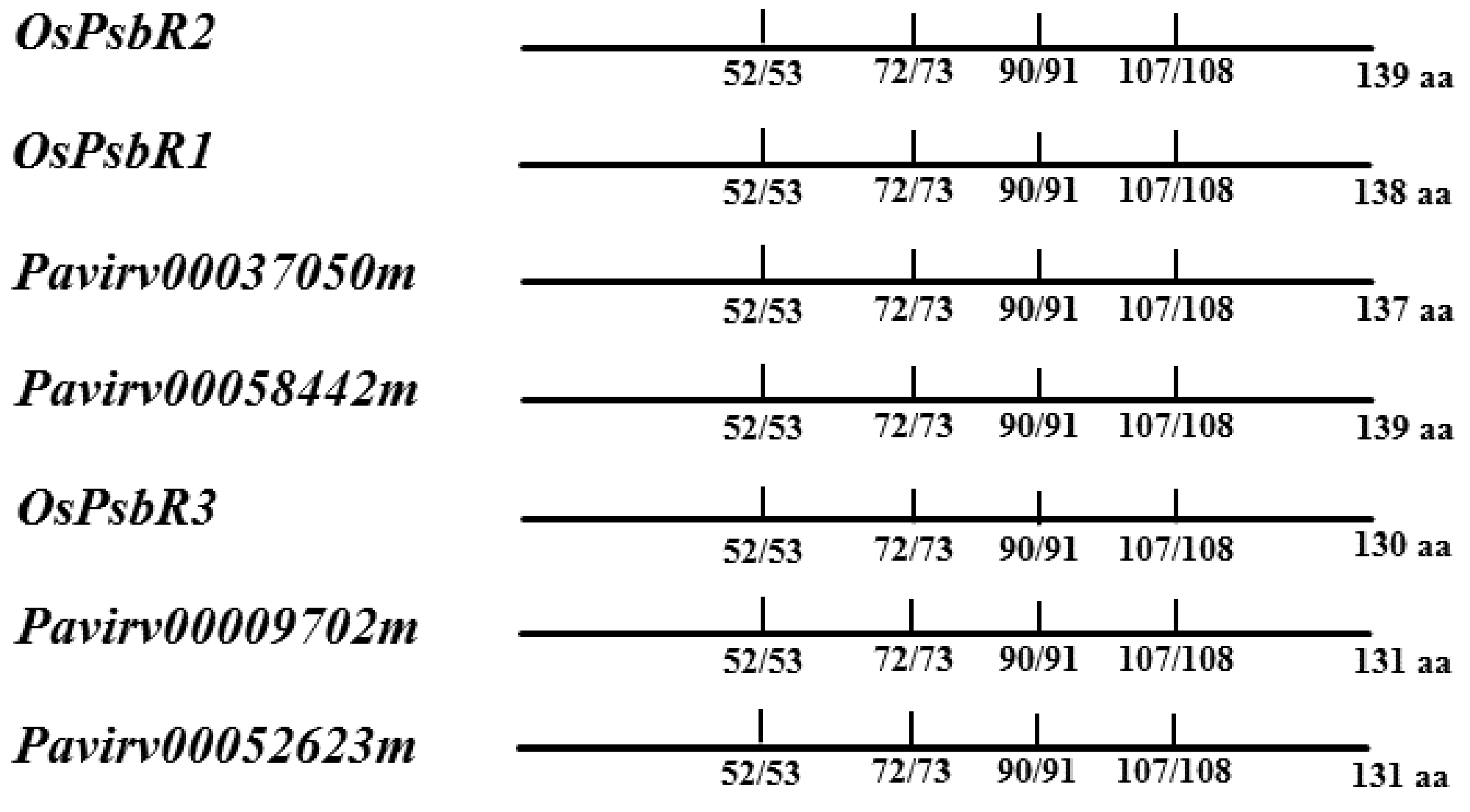


**Figure S7. The *in silico* expression profiles of the unitranscript entries of the potential switchgrass homologs of *OsLhcb1-1*, *OsLhcb1-2* and *OsLhcb2-1*, whose International Rice Genome Sequencing Project (IRGSP) gene IDs are Os09g17740 [54,55,57], Os1g41710 [54] and Os03g39610 [55], respectively, in different tissues of non-transformed switchgrass.** The unitranscript entry of each switchgrass homolog was obtained by using the cDNA sequence of each switchgrass homolog as the query sequence to blast the Noble Foundation switchgrass gene expression atlas PviUT V1.2 (<http://switchgrassgenomics.noble.org/>), and the *in silico* expression profiles were obtained by searching the gene expression atlas PviGEA for each unitranscript entry. The promoter region of *Pavirv00047797m* was used in the present study. Unitranscript entries: AP13CTG19188, Pavirv00047797m and *Pavirv00024895m*; KanlCTG01132, Pavirv00033469m; AP13ISTG52988, Pavirv00029871m and *Pavirv00032025m*; AP13ISTG54760, Pavirv00033114m and *Pavirv00055005m*; *KanlCTG05874-RC*, *Pavirv00027454m*; KanlCTG01647, Pavirv00069117m; *KanlCTG01924*, *Pavirv00026247m*; KanlCTG00765, Pavirv00057592m. E4-root, whole root system of E4 stage plant; E4-crown, whole crown of E4 stage plant; E4-node, pooled nodes of E4 the tiller; E4-LFB, pooled leaf blade from E4 tiller; E4-LSH, pooled leaf sheath of the E4 tiller; E4i3m, middle 1/5 fragment of internode 3; E4i3mVB, vascular bundle isolated from 1/5 fragment of internode 3; E4i4t, top 1/5 fragment of internode 4; E4i4m, middle 1/5 fragment of internode 4; E4i4b, bottom 1/5 fragment of internode 4; Inflo-meristem, inflorescence of rachis, primary and secondary branch meristem initiation stages 0.5-3.0 mm; Inflo-floret, inflorescence of glume and floret development stages 10-20 mm; Inflo-REL, inflorescence 50-150 mm; Inflo-PEM, inflorescence >200 mm. AP13seed0dap, whole flowers.


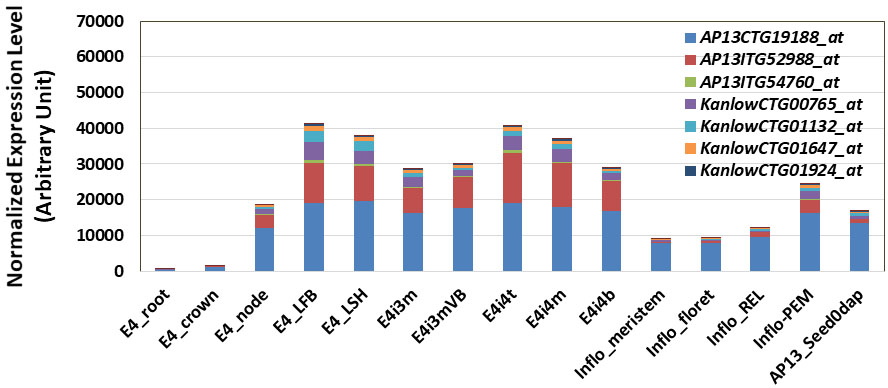


**Figure S8. The *in silico* expression profiles of the unitranscript entries of the potential switchgrass homologs of *Osppc1*, *2a*, *2b*, *3* and *4*, whose International Rice Genome Sequencing Project (IRGSP) gene IDs are Os02g0244700, Os08g0366000, Os09g0315700, Os01g0758300, and Os01g0208700, respectively [56], in different tissues of non-transformed switchgrass.** The unitranscript entry of each switchgrass homolog was obtained by using the cDNA sequence of each switchgrass homolog as the query sequence to blast the Noble Foundation switchgrass gene expression atlas PviUT V1.2 (<http://switchgrassgenomics.noble.org/>), and the *in silico* expression profiles were obtained by searching the gene expression atlas PviGEA for each unitranscript entry. The promoter region of *Pavirv00033161m* was used in the present study. Unitranscript entries: *KanlCTG00012*, *Pavirv00033161m*; *KanlCTG00243-RC*, *Pavirv00021858m*; *AP13CTG01078*, *Pavirv00041637m*; *AP13CTG04322*, *Pavirv00062625m*; *AP13CTG02943*, *Pavirv00007520m* and *Pavirv00055771m*; *AP13CTG16414*, *Pavirv00003818m* and *Pavirv00011136m*. E4-root, whole root system of E4 stage plant; E4-crown, whole crown of E4 stage plant; E4-node, pooled nodes of E4 the tiller; E4-LFB, pooled leaf blade from E4 tiller; E4-LSH, pooled leaf sheath of the E4 tiller; E4i3m, middle 1/5 fragment of internode 3; E4i3mVB, vascular bundle isolated from 1/5 fragment of internode 3; E4i4t, top 1/5 fragment of internode 4; E4i4m, middle 1/5 fragment of internode 4; E4i4b, bottom 1/5 fragment of internode 4; Inflo-meristem, inflorescence of rachis, primary and secondary branch meristem initiation stages 0.5-3.0 mm; Inflo-floret, inflorescence of glume and floret development stages 10-20 mm; Inflo-REL, inflorescence 50-150 mm; Inflo-PEM, inflorescence >200 mm. AP13seed0dap, whole flowers.


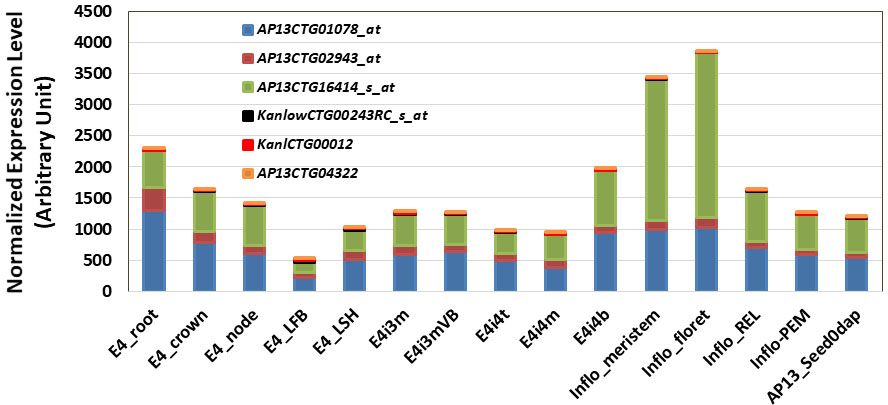


**Figure S9. The *in silico* expression profiles of the unitranscript entries of the potential switchgrass homologs of *OsPsbR1, 2* and *3*, whose International Rice Genome Sequencing Project (IRGSP) gene IDs are Os07g05360, Os07g05365, and Os08g10020, respectively [53], in different tissues of non-transformed switchgrass.** The unitranscript entry of each switchgrass homolog was obtained by using the cDNA sequence of each switchgrass homolog as the query sequence to blast the Noble Foundation switchgrass gene expression atlas PviUT V1.2 (<http://switchgrassgenomics.noble.org/>), and the *in silico* expression profiles were obtained by searching *the* gene expression atlas PviGEA for each unitranscript entry. The promoter region of *Pavirv00009702m* was used in the present study. Unitranscript entries: *AP13CTG22646*, *Pavirv00037050m* and *Pavirv00058442m*; AP13CTG07332, *Pavirv00009702m*; *AP13CTG19184*, *Pavirv00052623m*. E4-root, whole root system of E4 stage plant; E4-crown, whole crown of E4 stage plant; E4-node, pooled nodes of E4 the tiller; E4-LFB, pooled leaf blade from E4 tiller; E4-LSH, pooled leaf sheath of the E4 tiller; E4i3m, middle 1/5 fragment of internode 3; E4i3mVB, vascular bundle isolated from 1/5 fragment of internode 3; E4i4t, top 1/5 fragment of internode 4; E4i4m, middle 1/5 fragment of internode 4; E4i4b, bottom 1/5 fragment of internode 4; Inflo-meristem, inflorescence of rachis, primary and secondary branch meristem initiation stages 0.5-3.0 mm; Inflo-floret, inflorescence of glume and floret development stages 10-20 mm; Inflo-REL, inflorescence 50-150 mm; Inflo-PEM, inflorescence >200 mm. AP13seed0dap, whole flowers.


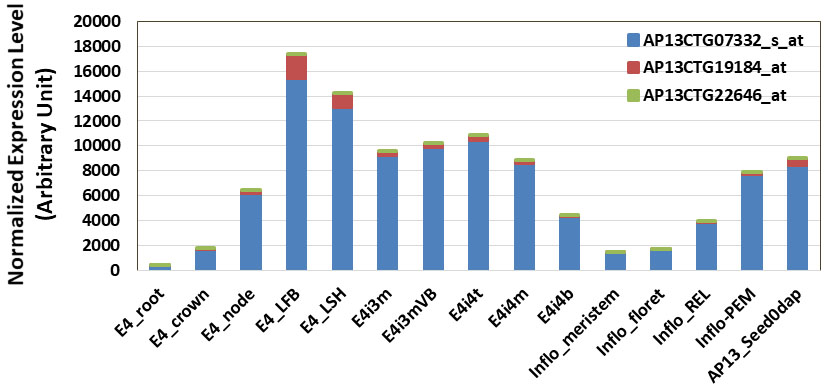


**Figure S10. The 764-bp-long promoter sequence of *PvLhcb* (i.e., *Pavirv00047797m*) used in the present study.** The forward and reverse primers used for PCR amplification and cloning of the promoter were underlined. The 5´UTR is indicated by blue font and the start codon, red font.

**
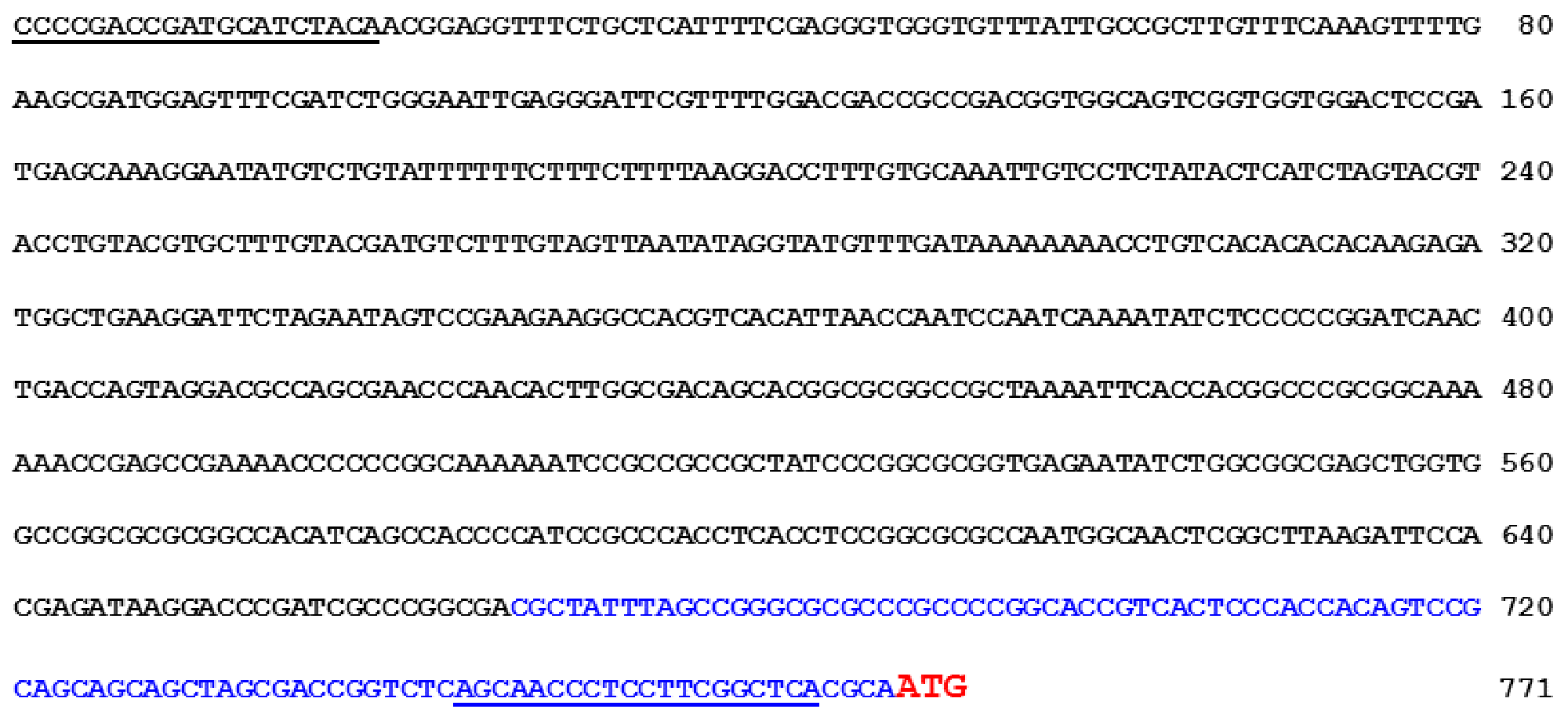
**

**Figure S11. The 1878-bp-long promoter sequence of *PvPEPC* (i.e., *Pavirv00033161m*) used in the present study.** The forward and reverse primers used for PCR amplification and cloning of the promoter were underlined. The 5´UTR sequence is indicated by blue font and the start codon is in red font. The start codon of its nearest upstream gene, which was reversely located, is indicated by orange font with the 5´UTR being in purple font.

**
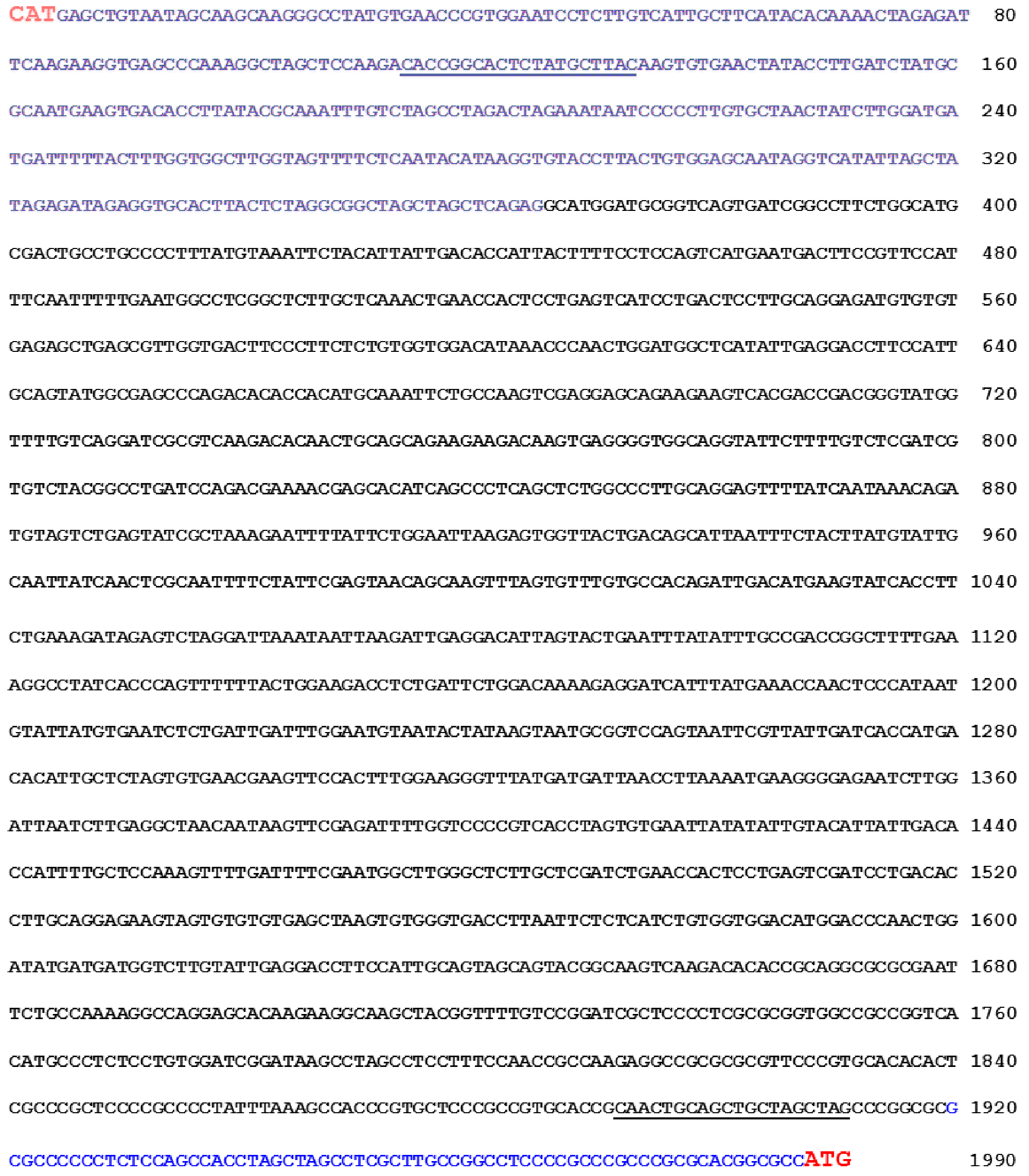
**

**Figure S12. The 2009-bp-long promoter sequence of *PvPsbR* (i.e., *Pavirv00009702m*) used in the present study.** The forward and reverse primers used for PCR amplification and cloning of the promoter were underlined. The 5´UTR sequence is indicated by blue font and the start codon is in the red font.

**
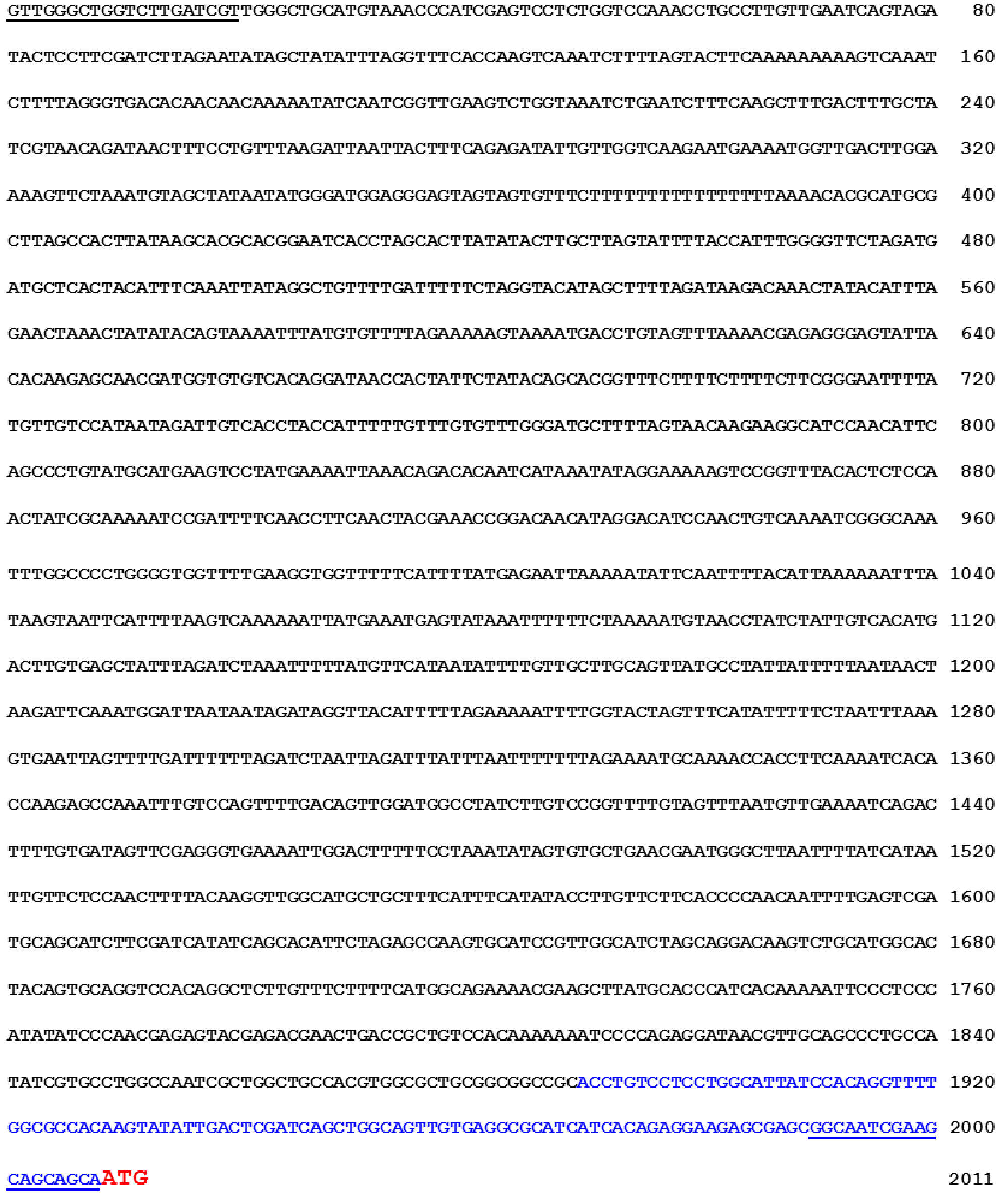
**

**Figure S13. Quantitative fluorometric GUS analysis of leaf blade, leaf sheath, stem and panicles of T0 stable transgenic rice containing each serial deletion of the *PvLhcb* promoter at the heading stage.** The rice plants were grown in the greenhouse at 25-29°C under 12 h photoperiods (390 μE m^−2^ s^−1^). Stable transgenic rice containing the promoter of *CaMV 35S* was used as positive control. The fluorometric GUS assay was conducted on at least 10 transgenic lines at the heading stage with the representatives being shown.


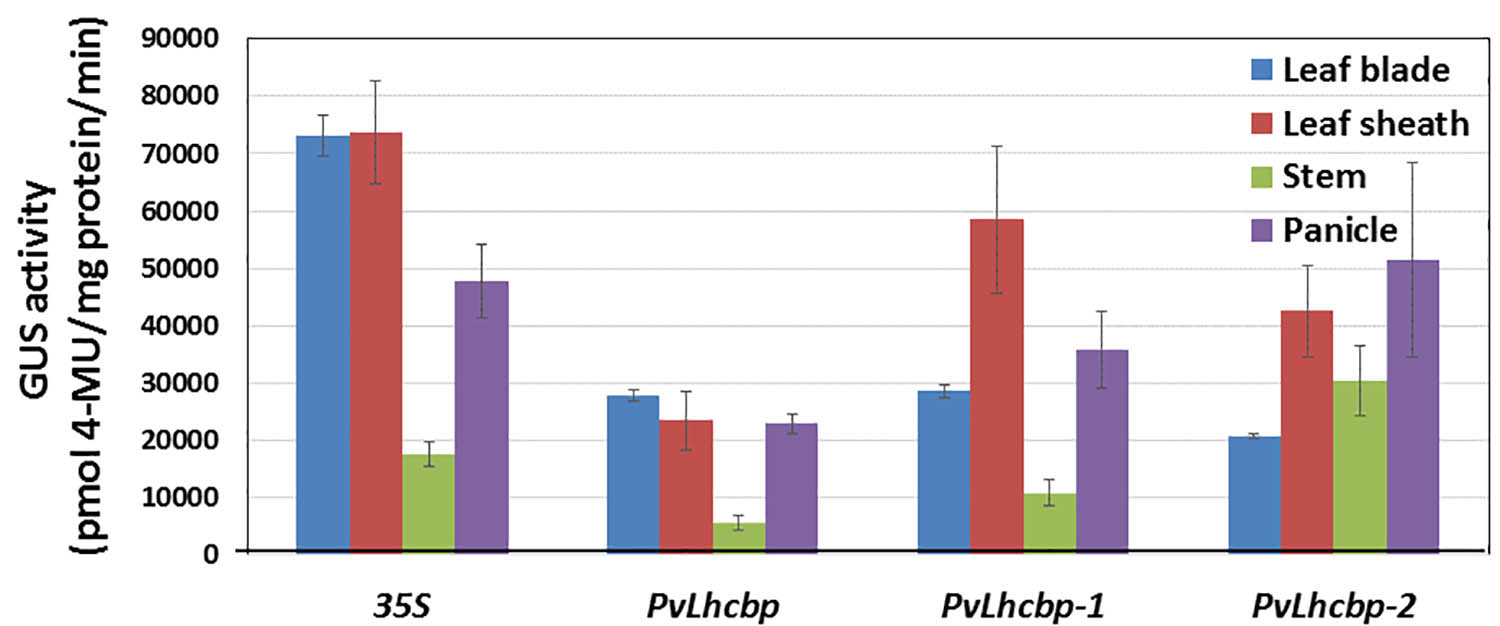


**Figure S14. Quantitative fluorometric GUS analysis of leaf blade, leaf sheath, stem and panicles of T0 stable transgenic rice containing each serial deletion of the *PvPEPC* promoter at the heading stage.** The rice plants were grown in the greenhouse at 25-29°C under 12 h photoperiods (390 μE m^−2^ s^−1^). Stable transgenic rice containing the promoter of *CaMV 35S* was used as positive control. The fluorometric GUS assay was conducted on at least 10 transgenic lines at the heading stage with the representatives being shown.


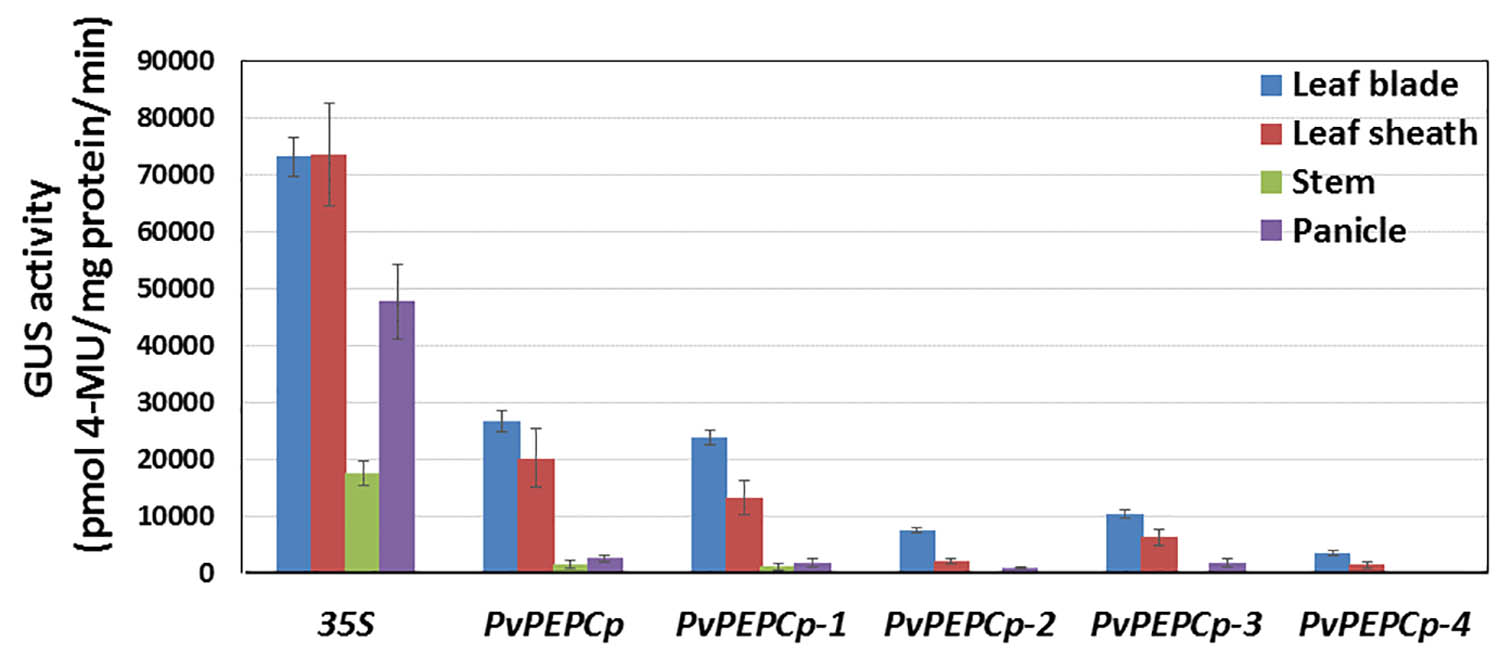

Supplement: Supplementary file 1 — Additional file 1: Table S1. Sugars (g/g CWR) released by enzymatic hydrolysis from the transgenic switchgrass lines expressing PvMYB4 under the control of each of the three green tissue-specific promoters. Figure S1. Comparison of the deduced amino acid sequences of the rice Lhcb genes and their homologs in switchgrass. Figure S2. Comparison of the deduced amino acid sequences of the rice PEPC gene and its homologs in switchgrass. Figure S3. Comparison of the deduced amino acid sequences of the rice PsbR genes and their homologs in switchgrass. Figure S4. The gene structures of the three rice Lhcb genes (i.e., OsLhcb1-1, OsLhcb1-2, and OsLhcb2-1, whose International Rice Genome Sequencing Project (IRGSP) gene IDs are Os09g17740 [54, 55, 57], Os1g41710 [54], and Os03g39610 [55], respectively) and their switchgrass homologs with the highest amino acid sequence similarities. Figure S5. The gene structures of the five plant-type rice PEPC genes (i.e., Osppc1, 2a, 2b, 3, and 4, whose International Rice Genome Sequencing Project (IRGSP) gene IDs are Os02g0244700, Os08g0366000, Os09g0315700, Os01g0758300, and Os01g0208700, respectively [56]) and their switchgrass homologs with the highest amino acid sequence similarities. Figure S6. The gene structures of the three rice PsbR genes (i.e., OsPsbR1, 2 and 3, whose International Rice Genome Sequencing Project (IRGSP) gene IDs are Os07g05360, Os07g05365, and Os08g10020, respectively [53]) and their switchgrass homologs with the highest amino acid sequence similarities. Figure S7. The in silico expression profiles of the unitranscript entries of the potential switchgrass homologs of OsLhcb1-1, OsLhcb1-2, and OsLhcb2-1, whose International Rice Genome Sequencing Project (IRGSP) gene IDs are Os09g17740 [54, 55, 57], Os1g41710 [54], and Os03g39610 [55], respectively, in different tissues of non-transformed switchgrass. Figure S8. The in silico expression profiles of the unitranscript entries of the potential switchgrass homologs o [file 13068_2018_1119_MOESM1_ESM.doc]
